# Supplementary material for: Uptake of best practice recommendations in the management of patients with diabetes and periodontitis: a cross-sectional survey of healthcare professionals in primary care
Source: BMJ Open. 2020 Jan 30;10(1):e032369. doi: 10.1136/bmjopen-2019-032369 (PMC7045148; doi:10.1136/bmjopen-2019-032369)
Supplement: Supplementary data [file bmjopen-2019-032369supp001.pdf]

Block 1

Survey completion instructions.

The **purpose of this survey** is to assess the clinical practices of healthcare professionals with regard to their patients with **poorly controlled diabetes** (HbA1c > 58mmol/mol or > 7.5%). **All surveys are confidential.**
If you manage patients with diabetes, you are invited to complete this survey. Completion will take **around 30 minutes, for which you will be remunerated** at your professional rate.
The survey has 21 questions separated into two sections: section 1 asks **6 questions** about you; and section 2 **(15 questions),** asks about the management of your patients with diabetes who are poorly controlled (HbA1c > 58mmol/mol or > 7.5%).
Please read each question carefully and answer it to the best of your ability. **There are no correct or incorrect responses**; we are merely interested in your point of view. The survey may appear to be repetitive since several of the statements are worded in a similar manner. It is the nature of this study that entails this methodological approach. We would **really appreciate your involvement**, as we wish to obtain **a 'snapshot' of current practice** in the UK regarding the dental management of people with diabetes.

Treating periodontitis (gum disease) can reduce HbA1c [4,5,6]

- Periodontitis impairs glycaemic control in people with diabetes1 and diabetes is a major risk factor for periodontitis (increasing the risk of periodontitis 3-fold).2

- A Cochrane systematic review reported a reduction in HbA1c of 4.4 mmol/mol (0.4 percentage points) 3-4 months after periodontal therapy,3 a clinical impact equivalent to that of many second-line pharmaceutical therapies for diabetes but without the risk of drug side-effects.

Aim of the research:

- The purpose of this survey is to find out the views and perspectives of healthcare professionals who manage patients with diabetes regarding the evidence supporting the links between diabetes and periodontitis.

References
1. Preshaw PM, Alba AL, Herrera D, Jepsen S, Konstantinidis A, Makrilakis K, et al. Periodontitis and diabetes: a two-way relationship. *Diabetologia* 2012;55:21-31.
2. Mealey BL, Oates TW, American Academy of P. Diabetes mellitus and periodontal diseases. *J Periodontol* 2006;77:1289-303.
3. Simpson TC, Needleman I, Wild SH, Moles DR, Mills EJ. Treatment of periodontal disease for glycaemic control in people with diabetes. *Cochrane Database Syst Rev* 2010:CD004714.
4. Borgnakke WS, Ylöstalo PV, Taylor GW, Genco RJ. Effect of periodontal disease on diabetes: systematic review of epidemiologic observational evidence. *J Clin Periodontol* 2013;40:S135-S52.
5. Janket SJ, Wightman A, Baird AE, Van Dyke TE, Jones JA. Does periodontal treatment improve glycemic control in diabetic patients? A meta-analysis of intervention studies. *J Dent Res* 2005;84:1154-59.
6. Darré L, Vergnes JN, Gourdy P, Sixou M. Efficacy of periodontal treatment on glycaemic control in diabetic patients: A meta-analysis of interventional studies. *Diabetes Metab* 2008;34:497-506.

Please click the arrows on the bottom right of the page to proceed.

Default Question Block

SECTION 1

Please answer these questions about yourself

Q1. Sex

Male ○ Female ○

Q2. Job

○ Community nurse
○ Diabetes nurse specialist
○ Diabetologist
○ Dietician
○ GP
○ GP with specialist interest in diabetes (with or without qualification)
○ GP with specialist interest in other area
○ Podiatrist
○ Practice nurse
○ Other (please specify)

Version 1.0

Q3. Age

- < 30
- 30 - 40
- 40 - 50
- 50 - 60
- > 60

Q4. Year of most recent professional qualification (yyyy - approximate is fine)?

Q5. How many patients with diabetes (either type 1 or 2) do you see in an average month?

Q6. Please use this space to clarify any responses or write any comments, if you would like to.

Block 2

SECTION 2

Please read each question carefully and answer it to the best of your ability.
There are no correct or incorrect responses; we are merely interested in your point of view.

Q7.
Thinking of **your last 10 patients with diabetes who you had a conversation with regarding their poor HbA1c levels (i.e. > 58 mmol/mol or > 7.5%).**

How many have you informed about the links between diabetes and periodontitis?

For example, if you inform everyone, then circle 10. If you inform some but not all, then chose a number which represents the proportion that you inform.

0	1	2	3	4	5	6	7	8	9	10

Q8.
Thinking of **your last 10 patients with diabetes who you had a conversation with regarding their poor HbA1c levels (i.e. > 58 mmol/mol or > 7.5%).**

How many have you ssuggested to go for a dental check-up?

For example, if you suggest it to everyone, then circle 10. If you inform some but not all, then chose a number which represents the proportion that you inform.

0	1	2	3	4	5	6	7	8	9	10

Version 1.0

Q9.

This question refers to **informing your patients with diabetes about the links between diabetes and periodontitis, during a conversation regarding their poor HbA1c levels (i.e. > 58 mmol/mol or > 7.5%).**

For each statement please **read the stem carefully first**, then select an answer that best suits your experience using the options.

For example, take a look at the stem and the first statement below and read them carefully. If you feel confident that you can inform your patient about the links between diabetes and periodontitis even when work is busy, then choose option 'Strongly Agree' or 'Agree'. If you feel that work being busy may affect your confidence in informing your patient about the links, then choose option 'Disagree' or 'Strongly Disagree'. If you are not sure, choose 'Neither agree nor disagree'. Now re-read the stem followed by the next statement, and so on.

Stem: I am confident that I can inform my patients with diabetes about the links between diabetes and periodontitis <u>EVEN WHEN</u>…

Statements:

	Strongly Disagree	Disagree	Neither Agree nor Disagree	Agree	Strongly Agree
... work is busy	○	○	○	○	○
... it is someone else's responsibility	○	○	○	○	○
... there is already too much to do	○	○	○	○	○
... it is not a priority for me	○	○	○	○	○
... it is not a priority for the patient	○	○	○	○	○
... I am not set up for it	○	○	○	○	○
... there are problems accessing dental services	○	○	○	○	○
... I am running late	○	○	○	○	○
... the patient has very few of their own teeth	○	○	○	○	○
... the patient seems to have good dental health	○	○	○	○	○
... my colleagues are doing the same	○	○	○	○	○
... my colleagues are not doing the same	○	○	○	○	○

Q10.

This question refers to **informing your patients with diabetes about the links between diabetes and periodontitis, during a conversation regarding their poor HbA1c levels (i.e. > 58 mmol/mol or > 7.5%).**

Stem: I am confident **that I can provide a suggestion to go for a dental check-up <u>*EVEN* WHEN</u>...**

Statements:

	Strongly Disagree	Disagree	Neither Agree nor Disagree	Agree	Strongly Agree
... work is busy	○	○	○	○	○
... it is someone else's responsibility	○	○	○	○	○
... there is already too much to do	○	○	○	○	○
... it is not a priority for me	○	○	○	○	○
... it is not a priority for the patient	○	○	○	○	○

Version 1.0

... I am not set up for it	○	○	○	○	○
... there are problems accessing dental services	○	○	○	○	○
... I am running late	○	○	○	○	○
... the patient has very few of their own teeth	○	○	○	○	○
... the patient seems to have good dental health	○	○	○	○	○
... my colleagues are doing the same	○	○	○	○	○
... my colleagues are not doing the same	○	○	○	○	○

Q11.

This question is about **informing your <u>poorly controlled patients with diabetes</u> (HbA1c > 58 mmol/mol or > 7.5%) about the links between diabetes and periodontitis.**

Please read the statements carefully and chose an appropriate option.

	OPTIONS							
	Strongly agree	Agree	Neither Agree nor Disagree	Disagree	Strongly Disagree	Not relevant to my role	Not relevant at this stage	Not relevant to diabetes care
Statement 1: I can see how informing patients about the links between diabetes and periodontitis differs from my usual way of working	○	○	○	○	○	○	○	○
Statement 2: Staff in this organisation have a shared understanding of the purpose of informing patients about the links between diabetes and periodontitis	○	○	○	○	○	○	○	○
Statement 3: I understand how informing patients about the links between diabetes and periodontitis affects the nature of my own work	○	○	○	○	○	○	○	○
Statement 4: I can see the potential value of informing patients about the links between diabetes and periodontitis for my work	○	○	○	○	○	○	○	○
Statement 5: I believe that informing patients about the links between diabetes and periodontitis is a legitimate part of my role	○	○	○	○	○	○	○	○

Q12.

This question relates to **suggesting that your patients with HbA1c > 58 mmol/mol or > 7.5%, should go for a dental check-up.**

Please read the statements carefully and chose an appropriate option.

	OPTIONS							
	Strongly agree	Agree	Neither Agree nor Disagree	Disagree	Strongly Disagree	Not relevant to my role	Not relevant at this stage	Not relevant to diabetes care
Statement 1: I can see how suggesting that your patients with HbA1c > 58 mmol/mol or > 7.5%, should go for a dental check-up differs from my usual way of working	○	○	○	○	○	○	○	○
Statement 2: Staff in this organisation have a shared understanding of the purpose of suggesting that your patients with HbA1c > 58 mmol/mol or > 7.5%, should go for a dental check-up	○	○	○	○	○	○	○	○
Statement 3: I understand how suggesting that your patients with HbA1c > 58 mmol/mol or > 7.5%, should go for a dental check-up affects the nature of my own work	○	○	○	○	○	○	○	○
Statement 4: I can see the potential value of suggesting								

Version 1.0

Bissett SM, *et al. BMJ Open* 2020; 10:e032369. doi: 10.1136/bmjopen-2019-032369

that your patients with HbA1c > 58 mmol/mol or > 7.5%, should go for a dental check-up for my work	○	○	○	○	○	○	○	○
Statement 5: I believe that suggesting that your patients with HbA1c > 58 mmol/mol or > 7.5%, should go for a dental check-up is a legitimate part of my role	○	○	○	○	○	○	○	○

Q13.
Informing them about the links between diabetes and periodontitis will help my poorly controlled patients with diabetes (HbA1c > 58 mmol/mol or > 7.5%)?

Not at all	A little	Moderately	Quite a bit	Extremely
○	○	○	○	○

Q14.
Suggesting that they go for a dental check-up will help my patients with poorly controlled diabetes (HbA1c > 58 mmol/mol or > 7.5%)?

Not at all	A little	Moderately	Quite a bit	Extremely
○	○	○	○	○

Q15.
Iinforming my patients with poorly controlled diabetes (HbA1c > 58 mmol/mol or > 7.5%) about the links between diabetes and periodontitis, will be a good use of my time?

Never	Rarely	Sometimes	Often	All of the Time
○	○	○	○	○

Q16.
Suggesting that my patients with poorly controlled diabetes (HbA1c > 58 mmol/mol or >7.5%) go for a dental check-up will be a good use of my time?

Never	Rarely	Sometimes	Often	All of the Time
○	○	○	○	○

Q17.
This question asks about the **timing** of **informing** patients about the links between diabetes and periodontitis and **suggesting** to have a dental check-up. Please read the question and tick as many options as you like.

OPTIONS:

QUESTIONS:

	**Q1**: When is a good time to inform patients about the links between diabetes and periodontitis?	**Q2**: When is a good time to suggest having a dental check-up?
Never	☐	☐
At time of periodontal diagnosis	☐	☐
At time of diabetes diagnosis	☐	☐
At their routine dental check-up appointment	☐	☐
Alongside discussion regarding their HbA1c	☐	☐
When the patient has poorly controlled diabetes	☐	☐
When the patient is facing being prescribed oral medication (or additional oral medication) for their diabetes	☐	☐
When the patient is facing being put onto insulin or other injectables	☐	☐
At their first appointment in the practice	☐	☐
When the periodontal condition deteriorates	☐	☐
Other	☐	☐

Version 1.0

Bissett SM, *et al. BMJ Open* 2020; 10:e032369. doi: 10.1136/bmjopen-2019-032369

Q18.
This question asks about the **timing** of **informing** patients about the links between diabetes and periodontitis and **suggesting** to have a dental check-up. Please read the question and tick as many options as you like.

OPTIONS - choose as many as you like

	No one	GP	GP with special interest in diabetes	Dietician	Diabetologist	Dentist	Dental hygienist/therapist	Other
Q1: Who **could** inform about the links between diabetes and periodontitis?	☐	☐	☐	☐	☐	☐	☐	☐
Q2: Who **should** inform about the links between diabetes and periodontitis?	☐	☐	☐	☐	☐	☐	☐	☐
Q3: Who **could** suggest having a dental check-up?	☐	☐	☐	☐	☐	☐	☐	☐
Q4: Who **should** suggest having a dental check-up?	☐	☐	☐	☐	☐	☐	☐	☐

Q19.
This question relates to **your next 10 patients with poorly controlled diabetes (HbA1c>58 mmol/mol or > 7.5%).**

How many do you intend to inform about the links between diabetes and periodontitis?

0 1 2 3 4 5 6 7 8 9 10

Q20.
This question relates to **your next 10 patients with poorly controlled diabetes (HbA1c>58 mmol/mol or > 7.5%).**

How many do you intend to suggest going for a dental check-up?

0 1 2 3 4 5 6 7 8 9 10

Q21. Please use this space to clarify any responses or write any comments, if you would like to.

Version 1.0

Bissett SM, *et al. BMJ Open* 2020; 10:e032369. doi: 10.1136/bmjopen-2019-032369
