# Supplementary material for: Uptake of best practice recommendations in the management of patients with diabetes and periodontitis: a cross-sectional survey of healthcare professionals in primary care
Source: BMJ Open. 2020 Jan 30;10(1):e032369. doi: 10.1136/bmjopen-2019-032369 (PMC7045148; doi:10.1136/bmjopen-2019-032369)
Supplement: Author's manuscript [file bmjopen-2019-032369.draft_revisions.pdf]

BMJ Open

BMJ Open is committed to open peer review. As part of this commitment we make the peer review history of every article we publish publicly available.

When an article is published we post the peer reviewers' comments and the authors' responses online. We also post the versions of the paper that were used during peer review. These are the versions that the peer review comments apply to.

The versions of the paper that follow are the versions that were submitted during the peer review process. They are not the versions of record or the final published versions. They should not be cited or distributed as the published version of this manuscript.

BMJ Open is an open access journal and the full, final, typeset and author-corrected version of record of the manuscript is available on our site with no access controls, subscription charges or pay-per-view fees (http://bmjopen.bmj.com).

If you have any questions on BMJ Open's open peer review process please email info.bmjopen@bmj.com

BMJ Open

Uptake of best practice recommendations in the management of patients with diabetes and periodontitis: a cross-sectional mixed methods survey of healthcare professionals in primary care

Journal:	*BMJ Open*
Manuscript ID	bmjopen-2019-032369
Article Type:	Research
Date Submitted by the Author:	18-Jun-2019
Complete List of Authors:	Bissett, Susan; University of Newcastle upon Tyne, School of Dental Sciences<br>Rapley, Tim; Northumbria University Department of Social Work and Communities, Social Work, Education and Community Wellbeing<br>Preshaw, Philip; National University of Singapore, National University Centre for Oral Health<br>Presseau , Justin; Ottawa Hospital Research Institute,
Keywords:	Diabetes, Periodontitis, Clinical behaviours, Best practice

SCHOLARONE™
Manuscripts

I, the Submitting Author has the right to grant and does grant on behalf of all authors of the Work (as defined in the below author licence), an exclusive licence and/or a non-exclusive licence for contributions from authors who are: i) UK Crown employees; ii) where BMJ has agreed a CC-BY licence shall apply, and/or iii) in accordance with the terms applicable for US Federal Government officers or employees acting as part of their official duties; on a worldwide, perpetual, irrevocable, royalty-free basis to BMJ Publishing Group Ltd ("BMJ") its licensees and where the relevant Journal is co-owned by BMJ to the co-owners of the Journal, to publish the Work in this journal and any other BMJ products and to exploit all rights, as set out in our licence.

The Submitting Author accepts and understands that any supply made under these terms is made by BMJ to the Submitting Author unless you are acting as an employee on behalf of your employer or a postgraduate student of an affiliated institution which is paying any applicable article publishing charge ("APC") for Open Access articles. Where the Submitting Author wishes to make the Work available on an Open Access basis (and intends to pay the relevant APC), the terms of reuse of such Open Access shall be governed by a Creative Commons licence – details of these licences and which Creative Commons licence will apply to this Work are set out in our licence referred to above.

Other than as permitted in any relevant BMJ Author's Self Archiving Policies, I confirm this Work has not been accepted for publication elsewhere, is not being considered for publication elsewhere and does not duplicate material already published. I confirm all authors consent to publication of this Work and authorise the granting of this licence.

For peer review only

Uptake of best practice recommendations in the management of patients with diabetes and periodontitis: a cross-sectional mixed methods survey of healthcare professionals in primary care

Susan M Bissett,[1] Tim Rapley,[2] Philip M Preshaw,[1,3] Justin Presseau.[4,5]

*1 School of Dental Sciences, Newcastle University, Newcastle upon Tyne, UK

2 Faculty of Health and Life Sciences, Northumbria University, Newcastle upon Tyne, UK

3 National University Centre for Oral Health, National University of Singapore, Singapore

4 School of Epidemiology and Public Health, University of Ottawa, Ottawa, Canada

5 School of Psychology, University of Ottawa, Ottawa, Canada

Keywords: Diabetes; periodontitis; clinical behaviours; best practice

*Correspondence to: Dr Susan Bissett;

Email: s.m.bissett@ncl.ac.uk

ORCiD ID (0000-0002-1997-3027)

Word count 4051

For peer review only - http://bmjopen.bmj.com/site/about/guidelines.xhtml

ABSTRACT

Objectives: To investigate the practices of healthcare professionals in relation to published best practice recommendations for the multidisciplinary management of people with diabetes and periodontitis, focusing on two clinical behaviours: informing patients about the links between diabetes and periodontitis, and suggesting patients with poorly controlled diabetes go for a dental check-up.

Design: Cross-sectional mixed methods design utilising online questionnaires to assess self-reported performance and constructs from Social Cognitive Theory (SCT) and Normalisation Process Theory (NPT).

Setting: Primary care medical practices (n=37) in North and South West England.

Participants: 96 general practitioners (GPs), 48 nurses and 21 healthcare assistants (HCAs).

Results: Participants reported little to no informing patients about the links between diabetes and periodontitis or suggesting that they go for a dental check-up. Regarding future intent, both GPs (7.60±3.38) and nurses (7.94±3.69) scored significantly higher than HCAs (4.29±5.07) for SCT proximal goals (intention) in relation to informing patients about the links ($p<0.01$); and nurses (8.56±3.12) scored significantly higher than HCAs (5.14±5.04) for suggesting patients go for a dental check-up ($p<0.001$). All professional groups agreed on the potential value of both behaviours, and nurses scored significantly higher than GPs for legitimation (conforms to perception of job role) in relation to informing (nurses 4.16±0.71; GPs 3.77±0.76) and suggesting (nurses 4.13±0.66; GPs 3.75±0.83) (both $p<0.01$). Perception of professional role emerged as a relevant theme from all professional groups with the suggestion that the behaviours appeared to align best to the nurse as diabetes educator.

Conclusions: Healthcare professionals, particularly nurses, value informing patients with diabetes about the links between diabetes and periodontitis and suggesting they go for a dental check-up, and perceive these behaviours to align with their role in the multidisciplinary management of people with diabetes.

For peer review only - http://bmjopen.bmj.com/site/about/guidelines.xhtml

STRENGTHS AND LIMITATIONS OF THIS STUDY

- Although best practice recommendations for the multidisciplinary management of patients with diabetes and periodontitis have been published, there is limited research on their uptake; this is the first study to explore practices of UK medical healthcare professionals in the context of such recommendations
- We achieved a high participation rate (80% at the practice level, and 76% at the participant level) from a range of healthcare professionals (general practitioners, nurses and healthcare assistants).
- We utilised a novel combination of Social Cognitive Theory and Normalisation Process Theory to evaluate current practice.
- The cross-sectional design precludes causal inference and direction of effect, but provides the first indication of factors associated with multidisciplinary management of people with diabetes and periodontitis in primary medical care.

For peer review only

For peer review only - http://bmjopen.bmj.com/site/about/guidelines.xhtml

INRODUCTION

Diabetes is a prevalent chronic non-communicable disease that has significant impacts on well-being and quality of life. Type 1 and type 2 diabetes are the principal categories, and in the UK as of 2019, there were 3.8 million people diagnosed with diabetes, of whom around 90% have type 2 diabetes. In addition, almost 1 million people are thought to be living with type 2 diabetes that is not yet diagnosed. This brings the estimated total number of people affected by diabetes to 4.7 million, or approximately one in 10 of the UK population over 40 years of age. This figure is predicted to reach 5.5 million by 2030.[1] Treatment of diabetes requires a life-long management strategy and varies in complexity given the multifactorial aetiology of the condition.

Periodontitis is also a prevalent chronic non-communicable disease. It is characterised by bacterially-driven inflammation in the tooth-supporting tissues that results in connective tissue destruction and alveolar bone resorption, leading, ultimately to tooth loss.[2] The dental plaque biofilm initiates and perpetuates the inflammation and an early sign of gingival inflammation (gingivitis) is bleeding, for example after brushing the teeth. Periodontitis is generally slowly progressing and painless, thus patients commonly present with advanced periodontitis that they were hitherto unaware of or ignored, even if they had been aware of some bleeding. Severe periodontitis has been reported to be the sixth-most prevalent disease in the world[3] and prevalence data in the UK have shown 8% of adults have advanced periodontitis.[4]

Periodontitis was initially identified as being a complication of diabetes in the early 1990s, the risk of periodontitis being increased by 2-3 times in a person with poorly controlled diabetes compared to individuals without.[5, 6] The pathogenic mechanisms linking periodontitis and diabetes are not yet fully understood but likely relate to upregulated systemic inflammation in each condition adversely affecting the other. The level of glycaemic control is key in determining risk,[7] and similar to the other complications of diabetes, the risk for periodontitis increases with poorer glycaemic control.[8-10]

Evidence supporting the potential to improve glycaemic control by treating periodontitis has emerged in more recent years, with several meta-analyses and two Cochrane reviews confirming reductions in HbA1c following effective periodontal therapy of up to 3-4 mmol/mol (0.3-0.4%) 3-4 months after periodontal treatment.[11-14] The precise mechanisms for the reduction of HbA1c following periodontal treatment are not completely

For peer review only - http://bmjopen.bmj.com/site/about/guidelines.xhtml

clear but likely arise from the combined effects of reduced inflammation and decreased bacterial challenge systemically, leading to a reduction in the systemic inflammatory state, and improvements in insulin resistance and insulin signalling.[10] Any reduction in HbA1c is important in people with diabetes, as it reduces the risk of diabetic complications. Every 1% reduction in HbA1c is associated with 21% reduced risk of any endpoint related to diabetes, 21% for deaths related to diabetes, 14% for myocardial infarction and 37% for microvascular complications.[15]

Over the years, a number of working groups have been established to provide guidance and recommendations for the multidisciplinary management of patients with periodontitis and diabetes. In 2007, the World Dental Federation (WDF) and the International Diabetes Federation (IDF) jointly organised a symposium on Oral Health and Diabetes. Experts agreed that there was urgent need to inform professionals, people with diabetes, policy makers and the public about the impact of diabetes on oral health; and they produced the IDF Guideline on Oral Health for People with Diabetes.[16] This guideline recommends that healthcare professionals should advise people with diabetes that good oral hygiene and regular dental checks are important and, in addition, adequate oral hygiene should be considered a normal part of diabetes self-management. In 2012, experts from the European Federation of Periodontology (EFP) and American Academy of Periodontology (AAP) also reviewed the evidence regarding the associations between diabetes and periodontitis.[17] They concluded that periodontitis was an independent predictor of several systemic conditions, including diabetes, and should be acknowledged as a major public health issue. Their manifesto stated that dental and medical communities should unite to develop a multidisciplinary approach to patient care.

Notwithstanding, previous research has shown that the academic and organisational silos in which dental and medical healthcare teams operate would appear to hinder shared knowledge and effective joint management of the two conditions.[18, 19] As knowledge alone is not sufficient to enable implementation of the delivery of healthcare interventions, research to identify the investment potential of healthcare professionals and enablers of change to aid implementation would be valued.

Accordingly, this study investigated the reported practices of healthcare professionals in relation to the management of diabetes and periodontitis to ascertain whether published best practice recommendations[16, 17] were being followed and to assess the factors which

For peer review only - http://bmjopen.bmj.com/site/about/guidelines.xhtml

For peer review only

predict behaviour, focusing on two recommended clinical behaviours: 1) Informing patients with diabetes about the links between diabetes and periodontitis; and 2) suggesting patients with poorly controlled diabetes go for a dental check-up.

METHODS

Design

The study used a cross-sectional design, involving online questionnaires (Qualtrics) to collect healthcare professionals' self-reported performance and views on the two clinical behaviours. The STROBE cross sectional reporting guidelines were followed.[20] As knowledge (or lack of knowledge) can influence cognitions towards behaviours, creating response bias,[21] we randomised (1:1 ratio) the provision (or not) of background information on the topic of the bidirectional relationship between diabetes and periodontitis as a preface at the start of the questionnaire, and the questionnaire was also piloted prior to use with healthcare professionals. As recommended by the UK Medical Research Council guidance for developing and evaluating complex interventions,[22] theory was used to explore healthcare professionals' behaviours in the context of diabetes and periodontitis, specifically a combination of Social Cognitive Theory (SCT)[23, 24] and Normalisation Process Theory (NPT).[25, 26]

SCT is a theory of motivation and action that describes key modifiable cognitions that can help to explain and improve the quality of care.[27-29] SCT holds that the care provided by healthcare professionals is a function of their self-efficacy (belief in their ability to provide the care), their outcome expectations (beliefs about the consequences of the care they provide), their proximal goals (intention to provide the care) and the present socio-structural determinants (external social and structural factors that act as barriers and enablers to care provision). NPT is an implementation theory used to identify, conceptualise and evaluate the factors that promote or inhibit the introduction, implementation and embedding of processes (such as patient management) into normal care.[30, 31] Subsequently, the NoMAD instrument[32, 33] was developed as a tool for using NPT to quantitatively assess implementation determinants, comprising four core constructs: coherence, cognitive participation, collective action, reflexive monitoring[30] and sub-constructs or items. The NoMAD tool can be customised by selecting sub-constructs as appropriate according to the study context (Table 1).[34]

For peer review only - http://bmjopen.bmj.com/site/about/guidelines.xhtml

Table 1 Definitions of Social Cognitive Theory (SCT) and Normalisation Process Theory (NPT) constructs utilised in this research

Social Cognitive Theory (SCT): a theory of motivation and action that is used to predict healthcare professionals' cognitions that may improve quality of care. SCT comprises 3 constructs:		
	Self-efficacy	The belief in one's ability to succeed in specific situations or accomplish a task.
	Outcome Expectations	One's expectations about the consequences of performing an action or behaviour.
	Proximal Goals	One's intention (i.e. motivation) that regulates future effort and action with respect to a particular behaviour.
Normalisation Process Theory (NPT): a framework that is used to evaluate the factors that promote or inhibit implementation of processes (such as specific aspects of patient management) into routine care. NPT comprises 4 core constructs:		
	Coherence	How healthcare professionals make sense of the behaviour or intervention, e.g. what it involves and why?
	Cognitive Participation	How healthcare professionals get involved and stay committed, e.g. can they see how they contribute?
	Collective Action	How healthcare professionals make it work in practice, e.g. what do they need to make it happen?
	Reflexive Monitoring	How healthcare professionals assess whether it is worth the effort, e.g. does it result in benefits to patient care?
NPT also includes up to 16 sub-constructs, and those that are relevant to the particular clinical scenario should be selected. We selected 5 NPT sub-constructs in this research, and the participants were asked to respond to these in the questionnaire:		
	Differentiation	I can see how the (behaviour) differs from usual ways of working.
	Communal specification	Staff in this organisation have a shared understanding of the purpose of this (behaviour).
	Individual specification	I understand how the (behaviour) affects the nature of my own work.
	Internalisation	I can see the potential value of the (behaviour) for my work.
	Legitimation	I believe that participating in the (behaviour) is a legitimate part of my role.

Table adapted from Bandura (SCT),[23, 24] May et al. and Finch et al. (NPT).[25, 26] Reproduced with permission from Bissett et al., British Dental Journal 2019;226:131-37.[34]

For peer review only

For peer review only - http://bmjopen.bmj.com/site/about/guidelines.xhtml

Measures

Self-reported past behaviour

The questionnaire measured past behaviour in relation to the last 10 patients with diabetes seen for whom the healthcare professionals reported performing any of the two recommended clinical behaviours (informing and suggesting). Response options ranged from 0 to 10 patients (i.e. the behaviour was performed on 'x' of their last 10 patients with diabetes) in order to simplify the estimation of that behaviour by the respondent, consistent with other studies that evaluated healthcare professionals' provision of diabetes-related healthcare.[35]

SCT constructs.

SCT constructs (self-efficacy, outcome expectations and proximal goals) were assessed using multi-item scales for both of the recommended clinical behaviours. Proximal goals was assessed on a 10-point scale to directly estimate for how many of the respondents' next 10 patients with diabetes they intended to engage in the behaviours. Self-efficacy and outcome expectations were also assessed for the two behaviours, using a 5-point Likert scale as follows: '1-strongly disagree', '2-disagree', '3-neither agree or disagree', '4-agree', and '5-strongly agree'. The wording of the items to assess SCT constructs was consistent with previous research.[35]

NPT constructs.

We customised the NoMAD tool to include five NPT sub-constructs: differentiation; communal specification; individual specification; internalisation; and legitimation.[31] All were measured using the same 5-point Likert scale as shown above. Multi-item questions were informed by our previous qualitative research in the context of the management of people with periodontitis and diabetes.[19]

The questionnaire also included a free-text box to permit respondents to provide any further comments for qualitative analysis.

Sample

Participants invited to complete the questionnaire included general practitioners (GPs), nurses and healthcare assistants (HCAs) working in primary care services involved in the care of people with diabetes. Participants were recruited via the UK Clinical Research Network (CRN), specifically North East and North Cumbria (NENC) CRN and South West Peninsula

For peer review only - http://bmjopen.bmj.com/site/about/guidelines.xhtml

(SWP) CRN. A network facilitator approached medical teams and gave them a study summary to consider. Expressions of interest were emailed to the researcher, who scheduled a telephone call to obtain a contact list of the staff members who managed people with diabetes. An email invitation containing a link to the questionnaire was then sent to each staff member. Respondents were given five weeks to complete and submit the questionnaire. During this time, two electronic reminders were sent as these have been shown to improve response:[36] one at three weeks following the initial invitation, and the other after four weeks, thus reminding the respondent that there was only one week left before the close of the survey. Completion and submission of the questionnaire was incentivised at half of the respondent's professional hourly salary, given the typical time to complete established during the pilot phase. The questionnaire responses were anonymous. An *a priori* sample size target of n=150 was set, consistent with thresholds suggested in systematic reviews of studies using constructs from behaviour theories to predict medical professional behaviour.[27, 37] The recruitment period ran from January to October 2016.

Statistical analysis

Statistical analysis was conducted using SPSS v23.0 for Windows. Summary sample characteristics and NPT data were calculated using descriptive analyses (means and standard deviations). The internal consistency of multi-item constructs was tested in order to combine results to a single mean score.[38, 39] Kruskal Wallis tests were used to identify significant differences in responses according to professional role (GPs, nurses, HCAs), with Mann Whitney post hoc tests with adjustment of the critical value of p as appropriate. SCT correlates of behaviour were assessed using binary univariate and multivariate logistic regression to identify construct predictors for each of the behaviours.

Qualitative analyses

Thematic analysis was used to identify common attributes within the data.[40, 41] Notable discussion points and specific comments of interest were identified and codes or key words were applied. Emergent patterns and resultant themes were formulated via an inductive approach. Quotes which illustrated concepts relating to a particular theme were considered in detail and unpacked to explore meaning and develop better understanding.

For peer review only - http://bmjopen.bmj.com/site/about/guidelines.xhtml

Ethical Approval

A favourable ethical opinion was obtained from North West-Greater Manchester West NHS Research Ethics Committee (16/NW/0030).

Patient and public involvement

Patients were involved with the design and development of this study. They helped to prioritise the objectives of the research.

RESULTS

The contact details of 46 medical practices were forwarded to the researcher by CRN facilitators: 11 from NENC CRN and 35 from SWP CRN. Of these, a total of 37 practices took part in the study (80% practice-level participation rate): 10 from NENC (27%) and 27 from SWP (73%). 176 questionnaires were returned from 217 that were sent out. Partially completed questionnaires were deleted list-wise to achieve a final sample of 165: 96 GPs, 48 nurses and 21 HCAs (76% participant-level response rate). Sample sociodemographic and clinical practice descriptive statistics (Table 2) show that the mean (± standard deviation) number of patients with a diabetes diagnosis was 6.3%±2.4%, and 67.6% of practices operated a separate diabetes clinic. The majority of the respondents were female (72%). HCAs saw a greater mean number of patients with diabetes per month (37.7±40.8), compared to GPs (33.2±31.8) and nurses (29.7±26.0), although the standard deviations were large.

For peer review only

For peer review only - http://bmjopen.bmj.com/site/about/guidelines.xhtml

Table 2 Sample characteristics of study population (n=165)

Practice level characteristics (n=37)		
Practice recruitment (N, %)	NENC	10 (27%)
	SWP	27 (73%)
List size (Min-Max)		3,600-35,818
Location (N, %)	Urban	7 (18.9%)
	Rural	11 (29.7%)
	Mixed	19 (51.4%)
Practices with separate diabetes clinic (N, %)		25 (67.6%)
% patient list >65 years (Mean ± SD)		22.5% ± 6.4%
% patient list have diabetes diagnosis (Mean ± SD)		6.3% ± 2.4%
Sample level characteristics (n=165)		
Sex (N, %)	Female	119 (72.1%)
	Male	46 (27.9%)
Age cohort (N, %)	< 30 years	5 (3.0%)
	30-40 years	39 (23.6%)
	40-50 years	58 (35.2%)
	50-60 years	56 (33.9%)
	> 60 years	7 (4.2%)
N patients with diabetes seen per month	GP (n=96)	33.2 ± 31.8
	Nurse (n=48)	29.7 ± 26.0
	HCA (n=21)	37.7 ± 40.8

Data for continuous variables presented as mean ± standard deviation.
%, percentage; GP, general practitioner; HCA, healthcare assistant.

For peer review only

For peer review only - http://bmjopen.bmj.com/site/about/guidelines.xhtml

Behaviour 1: Informing patients with diabetes about the links between diabetes and periodontitis

The questionnaire identified that healthcare professionals from all professional groups reported informing less than one of their last 10 patients with diabetes about the links between diabetes and periodontitis with no significant differences seen between GPs (0.23±0.69), nurses (0.58±1.81) and HCAs (0.24±0.63) (Table 3). The GPs' mean self-efficacy score (2.82±0.76) did not differ from that of HCAs (2.94±0.82), but was significantly lower than that of nurses (3.19±0.76) (p=0.01). The barriers perceived to undermine self-efficacy to inform patients included 'it is not a priority for the patient' and 'I am running late', whereas the highest ranked self-efficacy undermining barrier by all three professional groups was 'there are problems accessing dental services', with healthcare professionals scoring positively that they would remain confident to inform patients despite this challenge. The GPs' mean score (3.10±0.74) for outcome expectations did not differ significantly from that of HCAs (3.38±0.74), but was significantly lower than that of nurses (3.54±0.90) (p=0.01). For proximal goals (intention to inform) the responses were generally positive with GPs' mean score (7.60±3.38) not differing significantly from that of nurses (7.94±3.69); however, they were both significantly higher than that of HCAs (4.29±5.07) (p=0.01).

The NPT responses from all professional groups for differentiation indicated that this behaviour differed from usual ways of working with the GPs' mean response (4.06±0.89) not differing significantly from that of nurses (3.91±1.00) or HCAs (3.67±1.05). GPs' mean response score to communal specification (staff in the organisation share understanding of the purpose of informing) (2.27±0.83) did not differ from that of nurses (2.63±1.09) or HCAs (2.86±1.10). There were no significant differences detected for individual specification between GPs (3.40±0.93), nurses (3.30±0.95) and HCAs (3.07±0.70). Responses for internalisation and legitimation were positive. For internalisation (or seeing the potential value of informing), GPs' mean score (4.08±0.66) did not differ from that of nurses (4.26±0.61) or HCAs (4.07±0.59). The last cognitive participation item, 'I believe informing patients is a legitimate part of my role' was responded to positively. HCAs were the least positive (3.57±0.65) and significantly different to nurses (4.16±0.71), who also scored significantly higher than GPs (3.77±0.76) (p=0.01).

For peer review only - http://bmjopen.bmj.com/site/about/guidelines.xhtml

Table 3 Descriptive statistics of the two behaviours for SCT and NPT

**Behaviour**	**Job role**	**Past behaviour**	**Self-efficacy**	**Outcome expectations**	**Proximal goals**	**Differentiation**	**Communal specification**	**Individual specification**	**Internalisation**	**Legitimation**
Informing	GP (n=96)	0.23±0.69	2.82±0.76	3.10±0.74	7.60±3.38	4.06±0.89	2.27±0.83	3.40±0.93	4.08±0.66	3.77±0.76
	Nurse (n=48)	0.58±1.81	3.19±0.76	3.54±0.90	7.94±3.69	3.91±1.00	2.63±1.09	3.30±0.95	4.26±0.61	4.16±0.71
	HCA (n=21)	0.24±0.63	2.94±0.82	3.38±0.74	4.29±5.07	3.67±1.05	2.86±1.10	3.07±0.70	4.07±0.59	3.57±0.65
	p	0.95	**0.04** (GP vs Nurse **0.01**)	**0.01** (GP vs Nurse **0.01**)	**0.01** (GP vs HCA **0.01**) (Nurse vs HCA **0.01**)	0.29	**0.03**	0.25	0.32	**0.01** (GP vs Nurse **0.01**) (Nurse vs HCA **0.01**)
Suggesting	GP (n=96)	0.29±0.71	3.17±0.88	3.24±0.80	7.82±3.28	4.14±0.63	2.38±0.92	3.51±0.86	4.06±0.65	3.75±0.83
	Nurse (n=48)	1.10±2.46	3.54±0.78	3.58±0.88	8.56±3.12	3.93±1.10	2.84±1.04	3.35±0.90	4.24±0.60	4.13±0.66
	HCA (n=21)	0.14±0.48	3.15±0.71	3.55±0.82	5.14±5.04	3.73±0.88	3.27±1.03	3.21±0.70	3.87±0.52	3.62±0.65
	p	0.07	**0.02** (GP vs Nurse **0.01**)	0.81	**<0.001** (Nurse vs HCA **<0.001**)	0.25	**<0.001** (GP vs Nurse **0.01**) (GP vs HCA **<0.001**)	0.21	0.09	**0.01** (GP vs Nurse **0.01**)

Past behaviour and proximal goals were 10-point scales, i.e. for how many of the last 10 patients did the clinician perform the behaviour ('past behaviour'), and for how many of their next 10 patients does the clinician plan to perform the behaviour ('proximal goals'); the other measures were 5-point Likert scales: 1-strongly disagree', '2-disagree', '3-neither agree or disagree', '4-agree', and '5-strongly agree'.
Data presented as mean ± standard deviation.
p = test of differences between professional groups determined using Kruskal-Wallis (top level p value), with results of post-hoc Mann Whitney tests (with adjustment of critical value of p) presented in parentheses. GP, general practitioner; HCA, healthcare assistant; SCT, social cognitive theory; NPT, normalisation process theory. Statistically significant differences are indicated in bold font. Note: post-hoc analysis for communal specification (informing) did not identify statistically significant differences between groups following adjustment of critical value of p.

For peer review only - http://bmjopen.bmj.com/site/about/guidelines.xhtml

For peer review only

The SCT predictors for informing accounted for a small amount of variance (Cox & Snell R^2 0.05; Nagelkerke R^2 0.09) (Table 4). The covariate background information/no background information (OR=2.81; p=0.03) was statistically significant, indicating that it does appear to be associated with responses about informing patients about the links. Neither self-efficacy (OR=1.07, p=0.82), outcome expectations (OR=1.49, p=0.21) or proximal goals (OR=1.10, p=0.21) were significant predictors in a model that controlled for demographic factors and included other SCT constructs.

Behaviour 2: Suggesting patients with poorly controlled diabetes go for a dental check-up

All three professional groups reported suggesting patients with poorly controlled diabetes go for a dental check-up to one or less of the last 10 of their patients, with HCAs reporting the least amount (0.14±0.48), followed by GPs (0.29±0.71) and then nurses (1.10±2.46) (Table 3). The GPs' mean self-efficacy score (3.17±0.88) did not differ from that of HCAs (3.15±0.71), but was significantly lower than that of nurses (3.54±0.78) (p=0.01). The barriers that undermined self-efficacy to suggest patients go for a check-up most included 'it is not a priority for me/patient' and 'I am not set up for it', whereas the highest ranked item by all three professional groups was 'work being busy', with healthcare professionals scoring positively that they would remain confident to suggest patients with poorly controlled diabetes go for a check-up despite this challenge. GPs' mean score (3.24±0.80) for outcome expectations did not differ from that of HCAs (3.55±0.82) or nurses (3.58±0.88). For proximal goals (intention to suggest to go for a check-up), the responses were generally positive with the GPs' mean score (7.82±3.28) not differing from that of nurses (8.56±3.12), but the nurses' mean score was significantly higher than that of HCAs (5.14±5.04) (p<0.001).

The NPT responses for differentiation indicated that this behaviour was considered different from usual practice with GPs responding most strongly (4.14±0.63), but not significantly different to nurses (3.93±1.10) and HCAs (3.73±0.88). Whilst the GPs' mean response score to 'staff in the organisation share understanding of the purpose of suggesting' (2.38±0.92) was statistically significantly lower than that of nurses (2.84±1.04) (p=0.01) and HCAs (3.27±1.03) (p<0.001), for individual specification, there was no significant difference detected between GPs (3.51±0.86), nurses (3.35±0.90) and HCAs (3.21±0.70). Responses for internalisation and legitimation were positive. Nurses' mean score (4.24±0.60) did not differ from that of GPs (4.06±0.65) or HCAs (3.87±0.52) for seeing the potential value of suggesting to go for a dental check-up. For legitimation, nurses' mean score (4.13±0.66) did

For peer review only - http://bmjopen.bmj.com/site/about/guidelines.xhtml

not differ from that of HCAs (3.62±0.65), but was significantly higher than that of GPs (3.75±0.83) (p=0.01) for believing that suggesting patients go for a dental check-up was a legitimate part of their role.

The SCT predictors for suggesting patients go for a dental check-up accounted for a small amount of variance (Cox & Snell R^2 0.04; Nagelkerke R^2 0.07) (Table 4). The covariate background information/no background information (OR=1.26; p=0.57) was not associated with suggesting to go for a check-up, and outcome expectations (OR=1.06, p=0.81) and proximal goals (OR=0.99, p=0.90) were not statistically significant. Self-efficacy accounted for a statistically significant amount of variability (OR=1.71, p=0.04).

For peer review only

For peer review only - http://bmjopen.bmj.com/site/about/guidelines.xhtml

Table 4 Multivariate logistic regression model predicting past informing and suggesting (n=165)

Behaviours	Covariates and SCT Predictors	OR	SE	p	95% CI (B Coefficient)	
					Lower	Upper
Informing[1]	Background/no background	2.81	0.47	**0.03**	1.11	7.10
	Self-efficacy	1.07	0.30	0.82	0.60	1.92
	Outcome expectations	1.49	0.32	0.21	0.80	2.79
	Proximal goals	1.10	0.07	0.21	0.95	1.27
Suggesting[2]	Background/no background	1.26	0.40	0.57	0.58	2.74
	Self-efficacy	1.71	0.27	**0.04**	1.02	2.88
	Outcome expectations	1.06	0.26	0.81	0.64	1.75
	Proximal goals	0.99	0.06	0.90	0.88	1.12

p: statistically significant predictors indicated in bold font. OR, odds ratio; SE, standard error; CI confidence interval; B, exponential of β (odds ratio).
[1]Cox & Snell R^2 0.05, Nagelkerke R^2 0.09
[2]Cox & Snell R^2 0.04, Nagelkerke R^2 0.07

Qualitative responses to the two behaviours

Whilst the free text fields were optional, the overall mean response rate was 66%, indicating that two thirds of the respondents opted to provide further context, explanation of responses or opinion. The responses were broadly categorised into two themes 'Knowledge, skills and confidence' and 'Responsibility and role' (Table 5).

Generally, respondents had little to no knowledge about the bidirectional relationship between diabetes and periodontitis. Some respondents stated that they were grateful for the information and considered dissemination and incorporation of the newly acquired knowledge into their future practice (quote 1). For others, the response was not so positive and the lack of formal education was considered a barrier to informing their patients about the links. In addition, the idea of discussing the dental complications of diabetes was considered problematic and beyond professional scope of practice (quote 2).

The theme of responsibility and professional role featured in the responses with comments from all three professional groups. There was an assumption that staff with specialist diabetes training may already have knowledge of the bidirectional relationship between

For peer review only - http://bmjopen.bmj.com/site/about/guidelines.xhtml

diabetes and periodontitis, however, a lack of knowledge was not always seen to prohibit conversations with patients on the topic. One HCA indicated intention to inform patients about the links and suggest going for a dental check-up as they could signpost the patient to the nurse for more information (quote 3). The way that diabetes care is organised meant that some GPs considered that, given the complexities of managing patients with diabetes and other comorbidities, discussing oral health would not fit into their consultation appointments (quote 4). Notwithstanding, informing patients about the links and suggesting they get a check-up were felt to align with the diabetes review appointments. Self-reported comments from nurses further acknowledge their educational role in diabetes management and all professional groups considered these behaviours to be appropriate for a nurse to deliver in the context of diabetes education.

For peer review only

For peer review only - http://bmjopen.bmj.com/site/about/guidelines.xhtml

Table 5 Qualitative free text responses to informing patients with diabetes about the links between diabetes and periodontitis; and suggesting patients with poorly controlled diabetes go for a dental check-up.

Theme	Representative Quote
Knowledge, skills and confidence	1. Dental health is not something generally focused on in the GP practice and to be honest not something I have ever thought about. Obviously completing this [survey] will make me want to investigate further and suggesting dental care will now be added to the to-do list at review appointments. (Nurse)
	2. As I am a GP I stick to my area of expertise - I leave patients to make up their own minds about how much mouth care they want to pursue. (GP)
Responsibility and role	3. I will seek to find out more information from the trained nurses but as a healthcare assistant I will now mention the link and suggest a visit to the dentist but also direct them to the trained diabetic nurse for more information as I don't feel qualified enough to be giving a full explanation to patients. (HCA)
	4. I very rarely see people who specifically attend due to diabetes, or to discuss diabetic control. Nearly always they have multiple other concerns. It may be part of the diabetic review appointment (nurse does), but unless the patient has an oral health problem, I am unlikely to be able to make time in a consultation which could very well be for multiple other problems such as gynaecology or prostate. You could get the information included in preliminary diabetic information leaflets, and reinforce at specific review appointments. (GP)

For peer review only

For peer review only - http://bmjopen.bmj.com/site/about/guidelines.xhtml

DISCUSSION

An abundance of evidence links periodontitis and diabetes, including multiple meta-analyses and two Cochrane reviews which have confirmed the benefits of periodontitis treatment on diabetes control. Adding to a number of recommendation documents for clinical practice that have been published over the last decade or so, the EFP and IDF held a joint workshop in 2017 and produced outputs which were published simultaneously in the Journal of Clinical Periodontology and Diabetes Research and Clinical Practice,[42, 43] including guidelines for healthcare professionals, dental professionals and patients on the management of diabetes and periodontitis. Despite the existence of evidence and recommendations, the present research confirms that key clinical actions involved in enacting these recommendations are under-performed. The present research also identified modifiable correlates associated with variation in reported performance, suggesting targets for intervention to improve guideline recommendation uptake. Our cross sectional survey investigated the quantitative and qualitative self-reports of GPs, nurses and HCAs for two extant best practice clinical behaviours in relation to diabetes and periodontitis care published at the time of our research.[16, 17] These included informing patients about the links between periodontitis and diabetes and suggesting patients with poorly controlled diabetes go for a dental check-up.

Whilst SCT and NPT have previously been used effectively in health promotion in the context of chronic disease management,[44-46] the combination of these behavioural (SCT) and implementation (NPT) theories represents a novel approach to investigate clinicians' practices and behavioural correlates. Based on self-reported healthcare professionals' responses, there was little to no informing patients about the links between diabetes and periodontitis or suggesting that patients go for a dental check-up. All professional groups agreed that although both behaviours differed from usual ways of working (differentiation), there was potential value in the behaviours, indicated through positive responses for internalisation and intention (proximal goals).

We have previously reported on the practices of dental clinicians in relation to the management of patients with diabetes and periodontitis, again using a combination of SCT and NPT.[34] In that research, we investigated the uptake of three best practice recommendations for dental healthcare professionals; informing patients about the links between diabetes and periodontitis, considering the impact of periodontitis treatment on

For peer review only - http://bmjopen.bmj.com/site/about/guidelines.xhtml

glycaemic control, and contacting the patient's medical doctor. We identified that dental professionals were very likely to inform their patients about the links between the two diseases, and consider the impact of periodontitis on glycaemic control. However, there was very little evidence of dental clinicians contacting the patient's GP: periodontal specialists reported performing this behaviour in a minority of their patients with diabetes, and general dental practitioners and dental hygienists and therapists reported this behaviour in less than one of their last 10 patients. Respondents indicated that they would tend to communicate with the GP via the patient, as opposed to using direct communication or referral mechanisms. There was also evidence that some members of the dental team (e.g. dental hygienists and therapists) would not consider communicating with the patient's GP to be part of their job role. However, respondents indicated that they could see the potential value of this, and with appropriate training, an intervention to improve the uptake of this recommendation might particularly be targeted at dental hygienists and therapists, who see a higher number of patients with diabetes compared to other dental clinicians.

Similarly, in the present research, professional role emerged as a key theme in the qualitative free text data and appears to be important when considering the uptake and implementation of best practice recommendations. For both of the investigated behaviours, nurses' responses were significantly higher than those of GPs and HCAs for self-efficacy; and for proximal goals, GPs' and nurses' scores were statistically significantly higher than those of HCAs. Regarding legitimation, nurses scored statistically significantly higher than GPs for informing patients about the links; and significantly higher than GPs and HCAs for suggesting patients go for a dental check-up. Perhaps these responses reflect the pivotal role that a nurse often plays in patient education and health promotion in diabetes management.[47, 48]

Diabetes management can be complex and it is therefore no surprise that 'it is not a priority for me/patient' should be ranked as having the potential to undermine self-efficacy for both behaviours within this context, however problems associated with accessing NHS dental care and work being busy would not prevent the respondents from performing the behaviours. Previous research identified barriers to healthcare professionals talking to patients with diabetes about the oral complications of diabetes,[19, 49] but the present study would appear to indicate some investment potential which has not been reported in the literature before and which could improve the implementation of guidance. The low levels of current behaviours may explain the small amount of variance seen with the SCT predictors. Notwithstanding,

For peer review only - http://bmjopen.bmj.com/site/about/guidelines.xhtml

background information, whilst not a statistically significant predictor for suggesting to go for a check-up, was statistically significant for informing; and self-efficacy was a significant predictor for suggesting, and these should be considered as targets for training and implementation interventions.

CONCLUSION

Despite strong evidence and best practice recommendations, healthcare professionals report low levels of informing patients with diabetes about the links between diabetes and periodontitis and suggesting to go for a dental check-up. However, healthcare professionals, particularly nurses, value these behaviours and perceive them to be legitimate to their role as diabetes educators. Professional role appears to be an important factor when considering who, in the diabetes multidisciplinary team, is best aligned to speak to patients about diabetes and periodontitis.

For peer review only

For peer review only - http://bmjopen.bmj.com/site/about/guidelines.xhtml

Acknowledgements. We thank the participants who generously gave their time and the North East and North Cumbria (NENC) and South West Peninsula (SWP) Clinical Research Networks for facilitating recruitment.

Competing interests. None declared.

Funding. This research was funded by a UK National Institute for Health Research Doctoral Research Fellowship (DRF-2014-07-023) awarded to SMB.

Contribution statement. SMB, TR, PMP and JP designed the study. SMB recruited the participants and carried out the study. SMB, TR, PMP and JP analysed and interpreted the data. SMB wrote the manuscript and all authors revised it.

Data sharing statement. No additional data are available.

For peer review only

REFERENCES

1 Diabetes UK. Number of people with diabetes in UK. Available at: https://www.diabetes.org.uk/about_us/news/new-stats-people-living-with-diabetes/ Accessed 03/05/2019, 2019.

2 Preshaw PM, Alba AL, Herrera D, *et al.* Periodontitis and diabetes: a two-way relationship. *Diabetol* 2012;55:21-31.

3 Kassebaum NJ, Bernabé E, Dahiya M, *et al.* Global burden of severe periodontitis in 1990-2010: a systematic review and meta-regression. *J Dent Res* 2014;93:1045-53.

4 White D, Pitts N, Steele JG, *et al.* Diseases and related disorders. In: O'Sullivan I, editor. *Adult Dental Health Survey 2009*. London: NHS Information Centre for Health and Social Care, 2011.

5 Löe H. Periodontal disease: the sixth complication of diabetes mellitus. *Diabetes Care* 1993;16:329-34.

6 Mealey BL, Ocampo GL. Diabetes mellitus and periodontal disease. *Periodontol 2000* 2007;44:127-53.

7 Tsai C, Hayes C, Taylor GW. Glycemic control of type 2 diabetes and severe periodontal disease in the US adult population. *Community Dent Oral Epidemiol* 2002;30:182-92.

8 Pihlstrom BL, Michalowicz BS, Johnson NW. Periodontal diseases. *Lancet* 2005;366:1809-20.

9 Soskolne WA, Klinger A. The relationship between periodontal diseases and diabetes: an overview. *Ann Periodontol* 2001;6:91-8.

10 Polak D, Shapira L. An update on the evidence for pathogenic mechanisms that may link periodontitis and diabetes. *J Clin Periodontol* 2018;45:150-66.

11 Darré L, Vergnes JN, Gourdy P, *et al.* Efficacy of periodontal treatment on glycaemic control in diabetic patients: a meta-analysis of interventional studies. *Diabetes Metab* 2008;34:497-506.

For peer review only

For peer review only - http://bmjopen.bmj.com/site/about/guidelines.xhtml

12 Janket SJ, Wightman A, Baird AE*, et al.* Does periodontal treatment improve glycemic control in diabetic patients? A meta-analysis of intervention studies. *J Dent Res* 2005;84:1154-59.

13 Simpson TC, Needleman I, Wild SH*, et al.* Treatment of periodontal disease for glycaemic control in people with diabetes. *Cochrane Database Syst Rev* 2010:CD004714.

14 Simpson TC, Weldon JC, Worthington HV*, et al.* Treatment of periodontal disease for glycaemic control in people with diabetes mellitus. *Cochrane Database Syst Rev* 2015:CD004714.

15 Stratton IM, Adler AI, Neil HA*, et al.* Association of glycaemia with macrovascular and microvascular complications of type 2 diabetes (UKPDS 35): prospective observational study. *BMJ* 2000;321:405-12.

16 International Diabetes Federation. IDF Oral health for people with diabetes: guidelines. Available at: https://www.idf.org/e-library/guidelines/83-oral-health-for-people-with-diabetes.html Accessed 03/06/2019, 2009.

17 Chapple ILC, Genco R. Diabetes and periodontal diseases: consensus report of the Joint EFP/AAP Workshop on Periodontitis and Systemic Diseases. *J Clin Periodontol* 2013;40:S106-S12.

18 Larme AC, Pugh JA. Evidence-based guidelines meet the real world. The case of diabetes care. *Diabetes Care* 2001;24:1728-33.

19 Bissett SM, Stone KM, Rapley T*, et al.* An exploratory qualitative interview study about collaboration between medicine and dentistry in relation to diabetes management. *BMJ Open* 2013;3:e002192.

20 von Elm E AD, Egger M, Pocock SJ, Gotzsche PC, Vandenbroucke JP. . The Strengthening the Reporting of Observational Studies in Epidemiology (STROBE) Statement: guidelines for reporting observational studies.

21 Bandura A. *Self-efficacy: the Exercise of Control*. New York: Freeman, 1997.

22 Campbell M, Fitzpatrick R, Haines A*, et al.* Framework for design and evaluation of complex interventions to improve health. *BMJ* 2000;321:694-96.

For peer review only

For peer review only - http://bmjopen.bmj.com/site/about/guidelines.xhtml

23 Bandura A. Self-efficacy: toward a unifying theory of behavioral change. *Psychol Rev* 1977;84:191-215.

24 Bandura A. *Social Foundations of Thought and Action: a Social Cognitive Theory*. New Jersey, USA: Englewood Cliffs, 1986.

25 May C, Rapley T, Mair F*, et al.* Normalization process theory on-line users' manual, toolkit and NoMAD instrument. Available at: http://www.normalizationprocess.org Accessed 03/06/2019, 2015.

26 Finch TL, Girling M, May CR*, et al.* Nomad: implemetation measure based on normalization process theory. [Measurement instrument]. Available at: http://www.normalizationprocess.org Accessed 03/06/2019, 2015.

27 Godin G, Bélanger-Gravel A, Eccles M*, et al.* Healthcare professionals' intentions and behaviours: A systematic review of studies based on social cognitive theories. *Implement Sci* 2008;3:36.

28 Presseau J, Johnston M, Francis J*, et al.* Theory-based predictors of multiple clinician behaviors in the management of diabetes. *J Behav Med* 2014;37:607-20.

29 Stacey FG, James EL, Chapman K*, et al.* A systematic review and meta-analysis of social cognitive theory-based physical activity and/or nutrition behavior change interventions for cancer survivors. *J Cancer Surviv* 2015;9:305-38.

30 May C, Finch T, Mair F*, et al.* Understanding the implementation of complex interventions in health care: the normalization process model. *BMC Health Serv Res* 2007;7:148.

31 May C, Finch T. Implementation, embedding, and integration: an outline of normalization process theory. *Sociology* 2009;43:535-54.

32 Rapley T, Girling M, Mair FS*, et al.* Improving the normalization of complex interventions: part 1 - development of the NoMAD instrument for assessing implementation work based on normalization process theory (NPT). *BMC Med Res Methodol* 2018;18:133-33.

For peer review only - http://bmjopen.bmj.com/site/about/guidelines.xhtml

33 Finch TL, Girling M, May CR, *et al.* Improving the normalization of complex interventions: part 2 - validation of the NoMAD instrument for assessing implementation work based on normalization process theory (NPT). *BMC Med Res Methodol* 2018;18:135.

34 Bissett SM, Presseau J, Rapley T, *et al.* Uptake of best practice recommendations in the management of patients with diabetes and periodontitis: a cross-sectional survey of dental clinicians. *BDJ* 2019;226:131-37.

35 Eccles M, Hrisos S, Francis J, *et al.* Instrument development, data collection, and characteristics of practices, staff, and measures in the Improving Quality of Care in Diabetes (iQuaD) Study. *Implement Sci* 2011;6:61.

36 Clark L, Ronaldson S, Dyson L, *et al.* Electronic prompts significantly increase response rates to postal questionnaires: a randomized trial within a randomized trial and meta-analysis. *J Clin Epidemiol* 2015;68:1446-50.

37 Rashidian A, Miles J, Russell D, *et al.* Sample size for regression analyses of theory of planned behaviour studies: Case of prescribing in general practice. *Brit J Health Psych* 2006;11:581-93.

38 Cohen J. A power primer. *Psychol Bull* 1992;112:155-9.

39 Cronbach LJ. Coefficient alpha and the internal structure of tests. *Psychometrika* 1951;16:297-334.

40 Braun V, Clarke V. Using thematic analysis in psychology. *Qual Res Psychol* 2006;3:77-101.

41 Rapley T. Some pragmatics of qualitative data analysis. In: Silverman D, editor. *Qualitative Research*. 3rd ed. London: Sage Publications Ltd, 2011:273-90.

42 Sanz M, Ceriello A, Buysschaert M, *et al.* Scientific evidence on the links between periodontal diseases and diabetes: consensus report and guidelines of the Joint EFP/IDF Workshop on Periodontal Diseases and Diabetes. *Diabetes Res Clin Pract* 2018;137:231-41.

43 Sanz M, Ceriello A, Buysschaert M, *et al.* Scientific evidence on the links between periodontal diseases and diabetes: consensus report and guidelines of the Joint

For peer review only

For peer review only - http://bmjopen.bmj.com/site/about/guidelines.xhtml

EFP/IDF Workshop on Periodontal Diseases and Diabetes. *J Clin Periodontol* 2018;45:138-49.

44 Bandura A. Health promotion by social cognitive means. *Health Educ Behav* 2004;31:143-64.

45 Kennedy A, Bower P, Reeves D*, et al.* Implementation of self management support for long term conditions in routine primary care settings: cluster randomised controlled trial. *BMJ* 2013;346:f2882.

46 May CR, Cummings A, Girling M*, et al.* Using normalization process theory in feasibility studies and process evaluations of complex healthcare interventions: a systematic review. *Implement Sci* 2018;13:80.

47 Siminerio LM, Funnell MM, Peyrot M*, et al.* US nurses' perceptions of their role in diabetes care. *The Diabetes Educator* 2007;33:152-62.

48 Peimani M, Tabatabaei-Malazy O, Pajouhi M. Nurses' role in diabetes care: a review. *J Diabetes Metab Disord* 2010;9:4.

49 McDonald J, Jayasuriya R, Harris MF. The influence of power dynamics and trust on multidisciplinary collaboration: a qualitative case study of type 2 diabetes mellitus. *BMC Health Serv Res* 2012;12:63.

For peer review only

For peer review only - http://bmjopen.bmj.com/site/about/guidelines.xhtml

Reporting checklist for cross sectional study.

Based on the STROBE cross sectional guidelines.

Instructions to authors

Complete this checklist by entering the page numbers from your manuscript where readers will find each of the items listed below.

Your article may not currently address all the items on the checklist. Please modify your text to include the missing information. If you are certain that an item does not apply, please write "n/a" and provide a short explanation.

Upload your completed checklist as an extra file when you submit to a journal.

In your methods section, say that you used the STROBE cross sectionalreporting guidelines, and cite them as:

von Elm E, Altman DG, Egger M, Pocock SJ, Gotzsche PC, Vandenbroucke JP. The Strengthening the Reporting of Observational Studies in Epidemiology (STROBE) Statement: guidelines for reporting observational studies.

For peer review only

		Reporting Item	Page Number
**Title and abstract**			
Title	#1a	Indicate the study's design with a commonly used term in the title or the abstract	1

Abstract	#1b	Provide in the abstract an informative and balanced summary of what was done and what was found	2
**Introduction**			
Background / rationale	#2	Explain the scientific background and rationale for the investigation being reported	4
Objectives	#3	State specific objectives, including any prespecified hypotheses	5
**Methods**			
Study design	#4	Present key elements of study design early in the paper	6
Setting	#5	Describe the setting, locations, and relevant dates, including periods of recruitment, exposure, follow-up, and data collection	8
Eligibility criteria	#6a	Give the eligibility criteria, and the sources and methods of selection of participants.	8
	#7	Clearly define all outcomes, exposures, predictors, potential confounders, and effect modifiers. Give diagnostic criteria, if applicable	8
Data sources / measurement	#8	For each variable of interest give sources of data and details of methods of assessment (measurement). Describe comparability of assessment methods if there is more than one group. Give information separately for for exposed and unexposed groups if applicable.	8

For peer review only

Bias	#9	Describe any efforts to address potential sources of bias	6
Study size	#10	Explain how the study size was arrived at	9
Quantitative variables	#11	Explain how quantitative variables were handled in the analyses. If applicable, describe which groupings were chosen, and why	9
Statistical methods	#12a	Describe all statistical methods, including those used to control for confounding	9
Statistical methods	#12b	Describe any methods used to examine subgroups and interactions	9
Statistical methods	#12c	Explain how missing data were addressed	10
Statistical methods	#12d	If applicable, describe analytical methods taking account of sampling strategy	9
Statistical methods	#12e	Describe any sensitivity analyses	9
**Results**			
Participants	#13a	Report numbers of individuals at each stage of study—eg numbers potentially eligible, examined for eligibility, confirmed eligible, included in the study, completing follow-up, and analysed. Give information separately for for exposed and unexposed groups if applicable.	10
Participants	#13b	Give reasons for non-participation at each stage	10

For peer review only

Participants	#13c	Consider use of a flow diagram	11
Descriptive data	#14a	Give characteristics of study participants (eg demographic, clinical, social) and information on exposures and potential confounders. Give information separately for exposed and unexposed groups if applicable.	11
Descriptive data	#14b	Indicate number of participants with missing data for each variable of interest	10
Outcome data	#15	Report numbers of outcome events or summary measures. Give information separately for exposed and unexposed groups if applicable.	10
Main results	#16a	Give unadjusted estimates and, if applicable, confounder-adjusted estimates and their precision (eg, 95% confidence interval). Make clear which confounders were adjusted for and why they were included	13
Main results	#16b	Report category boundaries when continuous variables were categorized	13
Main results	#16c	If relevant, consider translating estimates of relative risk into absolute risk for a meaningful time period	n/a
Other analyses	#17	Report other analyses done—e.g., analyses of subgroups and interactions, and sensitivity analyses	16
**Discussion**			
Key results	#18	Summarise key results with reference to study objectives	19

For peer review only

Limitations	#19	Discuss limitations of the study, taking into account sources of potential bias or imprecision. Discuss both direction and magnitude of any potential bias.	3
Interpretation	#20	Give a cautious overall interpretation considering objectives, limitations, multiplicity of analyses, results from similar studies, and other relevant evidence.	20
Generalisability	#21	Discuss the generalisability (external validity) of the study results	19
**Other Information**			
Funding	#22	Give the source of funding and the role of the funders for the present study and, if applicable, for the original study on which the present article is based	22

The STROBE checklist is distributed under the terms of the Creative Commons Attribution License CC-BY. This checklist was completed on 13. June 2019 using https://www.goodreports.org/, a tool made by the EQUATOR Network in collaboration with Penelope.ai

For peer review only

BMJ Open

Uptake of best practice recommendations in the management of patients with diabetes and periodontitis: a cross-sectional survey of healthcare professionals in primary care

Journal:	*BMJ Open*
Manuscript ID	bmjopen-2019-032369.R1
Article Type:	Original research
Date Submitted by the Author:	04-Oct-2019
Complete List of Authors:	Bissett, Susan; University of Newcastle upon Tyne, School of Dental Sciences<br>Rapley, Tim; Northumbria University Department of Social Work and Communities, Social Work, Education and Community Wellbeing<br>Preshaw, Philip; National University of Singapore, National University Centre for Oral Health<br>Presseau , Justin; Ottawa Hospital Research Institute,
**Primary Subject Heading**:	Diabetes and endocrinology
Secondary Subject Heading:	Dentistry and oral medicine
Keywords:	Diabetes, Periodontitis, Clinical behaviours, Best practice

SCHOLARONE™
Manuscripts

I, the Submitting Author has the right to grant and does grant on behalf of all authors of the Work (as defined in the below author licence), an exclusive licence and/or a non-exclusive licence for contributions from authors who are: i) UK Crown employees; ii) where BMJ has agreed a CC-BY licence shall apply, and/or iii) in accordance with the terms applicable for US Federal Government officers or employees acting as part of their official duties; on a worldwide, perpetual, irrevocable, royalty-free basis to BMJ Publishing Group Ltd ("BMJ") its licensees and where the relevant Journal is co-owned by BMJ to the co-owners of the Journal, to publish the Work in this journal and any other BMJ products and to exploit all rights, as set out in our licence.

The Submitting Author accepts and understands that any supply made under these terms is made by BMJ to the Submitting Author unless you are acting as an employee on behalf of your employer or a postgraduate student of an affiliated institution which is paying any applicable article publishing charge ("APC") for Open Access articles. Where the Submitting Author wishes to make the Work available on an Open Access basis (and intends to pay the relevant APC), the terms of reuse of such Open Access shall be governed by a Creative Commons licence – details of these licences and which Creative Commons licence will apply to this Work are set out in our licence referred to above.

Other than as permitted in any relevant BMJ Author's Self Archiving Policies, I confirm this Work has not been accepted for publication elsewhere, is not being considered for publication elsewhere and does not duplicate material already published. I confirm all authors consent to publication of this Work and authorise the granting of this licence.

For peer review only

Uptake of best practice recommendations in the management of patients with diabetes and periodontitis: a cross-sectional survey of healthcare professionals in primary care

Susan M Bissett,[1] Tim Rapley,[2] Philip M Preshaw,[1,3] Justin Presseau.[4,5]

*1 School of Dental Sciences, Newcastle University, Newcastle upon Tyne, UK

2 Faculty of Health and Life Sciences, Northumbria University, Newcastle upon Tyne, UK

3 National University Centre for Oral Health, National University of Singapore, Singapore

4 School of Epidemiology and Public Health, University of Ottawa, Ottawa, Canada

5 School of Psychology, University of Ottawa, Ottawa, Canada

Keywords: Diabetes; periodontitis; clinical behaviours; best practice

*Correspondence to: Dr Susan Bissett;

Email: s.m.bissett@newcastle.ac.uk

ORCiD ID (0000-0002-1997-3027)

For peer review only

For peer review only - http://bmjopen.bmj.com/site/about/guidelines.xhtml

ABSTRACT

Objectives: To investigate the practices of healthcare professionals in relation to best practice recommendations for the multidisciplinary management of people with diabetes and periodontitis, focusing on two clinical behaviours: informing patients about the links between diabetes and periodontitis, and suggesting patients with poorly controlled diabetes go for a dental check-up.

Design: Cross-sectional design utilising online questionnaires to assess self-reported performance and constructs from Social Cognitive Theory (SCT) and Normalisation Process Theory (NPT).

Setting: Primary care medical practices (n=37) in North East, North Cumbria and South West of England Clinical Research Networks.

Participants: 96 general practitioners (GPs), 48 nurses and 21 healthcare assistants (HCAs).

Results: Participants reported little to no informing patients about the links between diabetes and periodontitis or suggesting that they go for a dental check-up. Regarding future intent, both GPs (7.60±3.38) and nurses (7.94±3.69) scored significantly higher than HCAs (4.29±5.07) for SCT proximal goals (intention) in relation to informing patients about the links ($p<0.01$); and nurses (8.56±3.12) scored significantly higher than HCAs (5.14±5.04) for suggesting patients go for a dental check-up ($p<0.001$). All professional groups agreed on the potential value of both behaviours, and nurses scored significantly higher than GPs for legitimation (conforms to perception of job role) in relation to informing (nurses 4.16±0.71; GPs 3.77±0.76) and suggesting (nurses 4.13±0.66; GPs 3.75±0.83) (both $p<0.01$). The covariate background information (OR=2.81; $p=0.03$) was statistically significant for informing patients about the links.

Conclusions: Despite evidence-informed best practice recommendations, healthcare professionals currently report low levels of informing patients with diabetes about the links between diabetes and periodontitis and suggesting patients go for a dental check-up. However, healthcare professionals, particularly nurses, value these behaviours and consider them appropriate to their role. Whilst knowledge of the evidence is important, future

For peer review only - http://bmjopen.bmj.com/site/about/guidelines.xhtml

guidelines should consider different strategies to enable implementation of the delivery of healthcare interventions.

For peer review only

For peer review only - http://bmjopen.bmj.com/site/about/guidelines.xhtml

STRENGTHS AND LIMITATIONS OF THIS STUDY

- Although best practice recommendations for the multidisciplinary management of patients with diabetes and periodontitis have been published, there is limited research on their uptake; this is the first study to explore practices of UK medical healthcare professionals in the context of such recommendations.
- We achieved a high participation rate (80% at the practice level, and 76% at the participant level) from a range of healthcare professionals (general practitioners, nurses and healthcare assistants); however, it is acknowledged that generalizability may be limited given that participants were recuited by convenience sampling from two geographically separate locations in the UK (the North East and North Cumbria and South West Peninsula Clinical Research Networks).
- We utilised a novel combination of Social Cognitive Theory and Normalisation Process Theory to evaluate current practice at an individual and organisational level.
- The cross-sectional design precludes causal inference and direction of effect, but provides the first indication of factors associated with multidisciplinary management of people with diabetes and periodontitis in UK primary medical care.

For peer review only

INRODUCTION

Diabetes is a prevalent chronic non-communicable disease that has significant impacts on well-being and quality of life. Type 1 and type 2 diabetes are the principal categories, and in the UK as of 2019, there were 3.8 million people diagnosed with diabetes, of whom around 90% have type 2 diabetes. In addition, almost 1 million people are thought to be living with type 2 diabetes that is not yet diagnosed. This brings the estimated total number of people affected by diabetes to 4.7 million, or approximately one in 10 of the UK population over 40 years of age. This figure is predicted to reach 5.5 million by 2030.[1] Treatment of diabetes requires a life-long management strategy and varies in complexity given the multifactorial aetiology of the condition.

Periodontitis is also a prevalent chronic non-communicable disease. It is characterised by bacterially-driven inflammation in the tooth-supporting tissues that results in connective tissue destruction and alveolar bone resorption, leading, ultimately to tooth loss.[2] The dental plaque biofilm initiates and perpetuates the inflammation and an early sign of gingival inflammation (gingivitis) is bleeding, for example after brushing the teeth. Periodontitis is generally slowly progressing and painless, thus patients commonly present with advanced periodontitis that they were hitherto unaware of or ignored, even if they had been aware of some bleeding. Severe periodontitis has been reported to be the sixth-most prevalent disease in the world[3] and prevalence data in the UK have shown 8% of adults have advanced periodontitis.[4]

Periodontitis was initially identified as being a complication of diabetes in the early 1990s, the risk of periodontitis being increased by 2-3 times in a person with poorly controlled diabetes compared to individuals without.[5, 6] The pathogenic mechanisms linking periodontitis and diabetes are not yet fully understood but likely relate to upregulated systemic inflammation in each condition adversely affecting the other. The level of glycaemic control is key in determining risk,[7] and similar to the other complications of diabetes, the risk for periodontitis increases with poorer glycaemic control.[8-10]

Evidence supporting the potential to improve glycaemic control by treating periodontitis has emerged in more recent years, with several meta-analyses and two Cochrane reviews confirming reductions in HbA1c following effective periodontal therapy of up to 3-4 mmol/mol (0.3-0.4%) 3-4 months after periodontal treatment.[11-14] The precise mechanisms for the reduction of HbA1c following periodontal treatment are not completely

For peer review only - http://bmjopen.bmj.com/site/about/guidelines.xhtml

clear but likely arise from the combined effects of reduced inflammation and decreased bacterial challenge systemically, leading to a reduction in the systemic inflammatory state, and improvements in insulin resistance and insulin signalling.[10] Any reduction in HbA1c is important in people with diabetes, as it reduces the risk of diabetic complications. Every 1% reduction in HbA1c is associated with 21% reduced risk of any endpoint related to diabetes, 21% for deaths related to diabetes, 14% for myocardial infarction and 37% for microvascular complications.[15]

Over the years, a number of working groups have been established to provide guidance and recommendations for the multidisciplinary management of patients with periodontitis and diabetes. In 2007, the World Dental Federation (WDF) and the International Diabetes Federation (IDF) jointly organised a symposium on Oral Health and Diabetes. Experts agreed that there was urgent need to inform professionals, people with diabetes, policy makers and the public about the impact of diabetes on oral health; and they produced the IDF Guideline on Oral Health for People with Diabetes.[16] This guideline recommends that healthcare professionals should advise people with diabetes that good oral hygiene and regular dental checks are important and, in addition, adequate oral hygiene should be considered a normal part of diabetes self-management. In 2012, experts from the European Federation of Periodontology (EFP) and American Academy of Periodontology (AAP) also reviewed the evidence regarding the associations between diabetes and periodontitis.[17] They concluded that periodontitis was an independent predictor of several systemic conditions, including diabetes, and should be acknowledged as a major public health issue. Their manifesto stated that dental and medical communities should unite to develop a multidisciplinary approach to patient care.

Notwithstanding, previous research has shown that the academic and organisational silos in which dental and medical healthcare teams operate would appear to hinder shared knowledge and effective joint management of the two conditions.[18, 19] As knowledge alone is not sufficient to enable implementation of the delivery of healthcare interventions, research to identify the investment potential of healthcare professionals and enablers of change to aid implementation would be valued.

Accordingly, this study investigated the reported practices of healthcare professionals in relation to the management of diabetes and periodontitis to ascertain whether published best practice recommendations[16, 17] were being followed and to assess the factors which

For peer review only - http://bmjopen.bmj.com/site/about/guidelines.xhtml

predict behaviour, focusing on two recommended clinical behaviours: 1) Informing patients with diabetes about the links between diabetes and periodontitis; and 2) suggesting patients with poorly controlled diabetes go for a dental check-up.

METHODS

Design

The study used a cross-sectional design, involving online questionnaires (Qualtrics) to collect healthcare professionals' self-reported performance and views on the two clinical behaviours. The STROBE cross sectional reporting guidelines were followed.[20] As knowledge (or lack of knowledge) can influence cognitions towards behaviours, creating response bias,[21] we randomised (1:1 ratio) the provision (or not) of background information on the topic of the bidirectional relationship between diabetes and periodontitis as a preface at the start of the questionnaire, and the questionnaire was also piloted prior to use with healthcare professionals. As recommended by the UK Medical Research Council guidance for developing and evaluating complex interventions,[22] theory was used to explore healthcare professionals' behaviours at an individual and organisational level in the context of diabetes and periodontitis, specifically a combination of Social Cognitive Theory (SCT)[23, 24] and Normalisation Process Theory (NPT).[25, 26] A sample of the survey is included as a supplementary file.

SCT is a theory of motivation and action that describes key modifiable cognitions that can help to explain and improve the quality of care.[27-29] SCT holds that the care provided by healthcare professionals is a function of their self-efficacy (belief in their ability to provide the care), their outcome expectations (beliefs about the consequences of the care they provide), their proximal goals (intention to provide the care) and the present socio-structural determinants (external social and structural factors that act as barriers and enablers to care provision). NPT is an implementation theory used to identify, conceptualise and evaluate the factors that promote or inhibit the introduction, implementation and embedding of processes (such as patient management) into normal care.[30, 31] Subsequently, the NoMAD instrument[32, 33] was developed as a tool for using NPT to quantitatively assess implementation determinants, comprising four core constructs: coherence, cognitive participation, collective action, reflexive monitoring[30] and sub-constructs or items. The

For peer review only - http://bmjopen.bmj.com/site/about/guidelines.xhtml

NoMAD tool can be customised by selecting sub-constructs as appropriate according to the study context (Table 1).[34]

For peer review only

For peer review only - http://bmjopen.bmj.com/site/about/guidelines.xhtml

Table 1 Definitions of Social Cognitive Theory (SCT) and Normalisation Process Theory (NPT) constructs utilised in this research

Social Cognitive Theory (SCT): a theory of motivation and action that is used to predict healthcare professionals' cognitions that may improve quality of care. SCT comprises 3 constructs:		
	Self-efficacy	The belief in one's ability to succeed in specific situations or accomplish a task.
	Outcome Expectations	One's expectations about the consequences of performing an action or behaviour.
	Proximal Goals	One's intention (i.e. motivation) that regulates future effort and action with respect to a particular behaviour.
Normalisation Process Theory (NPT): a framework that is used to evaluate the factors that promote or inhibit implementation of processes (such as specific aspects of patient management) into routine care. NPT comprises 4 core constructs:		
	Coherence	How healthcare professionals make sense of the behaviour or intervention, e.g. what it involves and why?
	Cognitive Participation	How healthcare professionals get involved and stay committed, e.g. can they see how they contribute?
	Collective Action	How healthcare professionals make it work in practice, e.g. what do they need to make it happen?
	Reflexive Monitoring	How healthcare professionals assess whether it is worth the effort, e.g. does it result in benefits to patient care?
NPT also includes up to 16 sub-constructs, and those that are relevant to the particular clinical scenario should be selected. We selected 5 NPT sub-constructs in this research, and the participants were asked to respond to these in the questionnaire:		
	Differentiation	I can see how the (behaviour) differs from usual ways of working.
	Communal specification	Staff in this organisation have a shared understanding of the purpose of this (behaviour).
	Individual specification	I understand how the (behaviour) affects the nature of my own work.
	Internalisation	I can see the potential value of the (behaviour) for my work.
	Legitimation	I believe that participating in the (behaviour) is a legitimate part of my role.

Table adapted from Bandura (SCT),[23, 24] May et al. and Finch et al. (NPT).[25, 26] Reproduced with permission from Bissett et al., British Dental Journal 2019;226:131-37.[34]

For peer review only

For peer review only - http://bmjopen.bmj.com/site/about/guidelines.xhtml

Measures

Self-reported past behaviour

The questionnaire measured past behaviour in relation to the last 10 patients with diabetes seen for whom the healthcare professionals reported performing any of the two recommended clinical behaviours (informing and suggesting). Response options ranged from 0 to 10 patients (i.e. the behaviour was performed on 'x' of their last 10 patients with diabetes) in order to simplify the estimation of that behaviour by the respondent, consistent with other studies that evaluated healthcare professionals' provision of diabetes-related healthcare.[35]

SCT constructs.

SCT constructs (self-efficacy, outcome expectations and proximal goals) were assessed using multi-item scales for both of the recommended clinical behaviours. Proximal goals was assessed on a 10-point scale to directly estimate for how many of the respondents' next 10 patients with diabetes they intended to engage in the behaviours. Self-efficacy and outcome expectations were also assessed for the two behaviours, using a 5-point Likert scale as follows: '1-strongly disagree', '2-disagree', '3-neither agree or disagree', '4-agree', and '5-strongly agree'. The wording of the items to assess SCT constructs was consistent with previous research.[35]

NPT constructs.

We customised the NoMAD tool to include five NPT sub-constructs: differentiation; communal specification; individual specification; internalisation; and legitimation.[31] All were measured using the same 5-point Likert scale as shown above. Multi-item questions were informed by our previous qualitative research in the context of the management of people with periodontitis and diabetes.[19]

Sample

Participants invited to complete the questionnaire included general practitioners (GPs), nurses and healthcare assistants (HCAs) working in primary care services involved in the care of people with diabetes. Participants were recruited via convenience sampling through the UK Clinical Research Network (CRN), specifically North East and North Cumbria (NENC) CRN and South West Peninsula (SWP) CRN. A network facilitator approached medical teams and gave them a study summary to consider. Expressions of interest were emailed to the

For peer review only

researcher, who scheduled a telephone call to obtain a contact list of the staff members who managed people with diabetes. An email invitation containing a link to the questionnaire was then sent to each staff member. Respondents were given five weeks to complete and submit the questionnaire. During this time, two electronic reminders were sent as these have been shown to improve response:[36] one at three weeks following the initial invitation, and the other after four weeks, thus reminding the respondent that there was only one week left before the close of the survey. Completion and submission of the questionnaire was incentivised at half of the respondent's professional hourly salary, given the typical time to complete established during the pilot phase. The questionnaire responses were anonymous. An *a priori* sample size target of n=150 was set, consistent with thresholds suggested in systematic reviews of studies using constructs from behaviour theories to predict medical professional behaviour.[27, 37] The recruitment period ran from January to October 2016.

Statistical analysis

Statistical analysis was conducted using SPSS v23.0 for Windows. Summary sample characteristics and NPT data were calculated using descriptive analyses (means and standard deviations). The internal consistency of multi-item constructs was tested in order to combine results to a single mean score.[38, 39] Kruskal Wallis tests were used to identify significant differences in responses according to professional role (GPs, nurses, HCAs), with Mann Whitney post hoc tests with adjustment of the critical value of p as appropriate. SCT correlates of behaviour were assessed using binary univariate and multivariate logistic regression to identify construct predictors for each of the behaviours.

Patient and Public Involvement

The researcher (SMB) attended regular (2-3 times a year) meetings of Newcastle School of Dental Sciences Patient and Public Involvment (PPI) group. The PPI members contributed at every stage of the research and the results were disseminated in the form of a presentation (SMB).

Ethics Approval

Approval was obtained from North West-Greater Manchester West NHS Research Ethics Committee (16/NW/0030). Site approval was granted from each practice principal who approached eligible staff members. Interested staff members were sent an email invitation to the survey and consented to participate by clicking the link and submitting the survey.

For peer review only - http://bmjopen.bmj.com/site/about/guidelines.xhtml

RESULTS

The contact details of 46 medical practices were forwarded to the researcher by CRN facilitators: 11 from NENC CRN and 35 from SWP CRN. Of these, a total of 37 practices took part in the study (80% practice-level participation rate): 10 from NENC (27%) and 27 from SWP (73%). 176 questionnaires were returned from 217 that were sent out. Reasons for non-participation were not known. Partially completed questionnaires were deleted list-wise to achieve a final sample of 165: 96 GPs, 48 nurses and 21 HCAs (76% participant-level response rate). Sample sociodemographic and clinical practice descriptive statistics (Table 2) show that the mean (± standard deviation) number of patients with a diabetes diagnosis was 6.3%±2.4%, and 67.6% of practices operated a separate diabetes clinic. The majority of the respondents were female (72%). HCAs saw a greater mean number of patients with diabetes per month (37.7±40.8), compared to GPs (33.2±31.8) and nurses (29.7±26.0), although the standard deviations were large.

For peer review only - http://bmjopen.bmj.com/site/about/guidelines.xhtml

Table 2 Sample characteristics of study population (n=165)

Practice level characteristics (n=37)		
Practice recruitment (N, %)	NENC	10 (27%)
	SWP	27 (73%)
List size (Min-Max)		3,600-35,818
Location (N, %)	Urban	7 (18.9%)
	Rural	11 (29.7%)
	Mixed	19 (51.4%)
Practices with separate diabetes clinic (N, %)		25 (67.6%)
% patient list >65 years (Mean ± SD)		22.5% ± 6.4%
% patient list have diabetes diagnosis (Mean ± SD)		6.3% ± 2.4%
Sample level characteristics (n=165)		
Sex (N, %)	Female	119 (72.1%)
	Male	46 (27.9%)
Age cohort (N, %)	< 30 years	5 (3.0%)
	30-40 years	39 (23.6%)
	40-50 years	58 (35.2%)
	50-60 years	56 (33.9%)
	> 60 years	7 (4.2%)
N patients with diabetes seen per month	GP (n=96)	33.2 ± 31.8
	Nurse (n=48)	29.7 ± 26.0
	HCA (n=21)	37.7 ± 40.8

Data for continuous variables presented as mean ± standard deviation.
%, percentage; GP, general practitioner; HCA, healthcare assistant.

For peer review only

For peer review only - http://bmjopen.bmj.com/site/about/guidelines.xhtml

Behaviour 1: Informing patients with diabetes about the links between diabetes and periodontitis

The questionnaire identified that healthcare professionals from all professional groups reported informing less than one of their last 10 patients with diabetes about the links between diabetes and periodontitis with no significant differences seen between GPs (0.23±0.69), nurses (0.58±1.81) and HCAs (0.24±0.63) (Table 3). The GPs' mean self-efficacy score (2.82±0.76) did not differ from that of HCAs (2.94±0.82), but was significantly lower than that of nurses (3.19±0.76) (p=0.01). The barriers perceived to undermine self-efficacy to inform patients included 'it is not a priority for the patient' and 'I am running late', whereas the highest ranked self-efficacy undermining barrier by all three professional groups was 'there are problems accessing dental services', with healthcare professionals scoring positively that they would remain confident to inform patients despite this challenge. The GPs' mean score (3.10±0.74) for outcome expectations did not differ significantly from that of HCAs (3.38±0.74), but was significantly lower than that of nurses (3.54±0.90) (p=0.01). For proximal goals (intention to inform) the responses were generally positive with GPs' mean score (7.60±3.38) not differing significantly from that of nurses (7.94±3.69); however, they were both significantly higher than that of HCAs (4.29±5.07) (p=0.01).

The NPT responses from all professional groups for differentiation indicated that this behaviour differed from usual ways of working with the GPs' mean response (4.06±0.89) not differing significantly from that of nurses (3.91±1.00) or HCAs (3.67±1.05). GPs' mean response score to communal specification (staff in the organisation share understanding of the purpose of informing) (2.27±0.83) did not differ from that of nurses (2.63±1.09) or HCAs (2.86±1.10). There were no significant differences detected for individual specification between GPs (3.40±0.93), nurses (3.30±0.95) and HCAs (3.07±0.70). Responses for internalisation and legitimation were positive. For internalisation (or seeing the potential value of informing), GPs' mean score (4.08±0.66) did not differ from that of nurses (4.26±0.61) or HCAs (4.07±0.59). The last cognitive participation item, 'I believe informing patients is a legitimate part of my role' was responded to positively. HCAs were the least positive (3.57±0.65) and significantly different to nurses (4.16±0.71), who also scored significantly higher than GPs (3.77±0.76) (p=0.01).

For peer review only - http://bmjopen.bmj.com/site/about/guidelines.xhtml

Table 3 Descriptive statistics of the two behaviours for SCT and NPT

Behaviour	Job role	Past behaviour	Self-efficacy	Outcome expectations	Proximal goals	Differentiation	Communal specification	Individual specification	Internalisation	Legitimation
Informing	GP (n=96)	0.23±0.69	2.82±0.76	3.10±0.74	7.60±3.38	4.06±0.89	2.27±0.83	3.40±0.93	4.08±0.66	3.77±0.76
	Nurse (n=48)	0.58±1.81	3.19±0.76	3.54±0.90	7.94±3.69	3.91±1.00	2.63±1.09	3.30±0.95	4.26±0.61	4.16±0.71
	HCA (n=21)	0.24±0.63	2.94±0.82	3.38±0.74	4.29±5.07	3.67±1.05	2.86±1.10	3.07±0.70	4.07±0.59	3.57±0.65
	p	0.95	**0.04** (GP vs Nurse **0.01**)	**0.01** (GP vs Nurse **0.01**)	**0.01** (GP vs HCA **0.01**) (Nurse vs HCA **0.01**)	0.29	**0.03**	0.25	0.32	**0.01** (GP vs Nurse **0.01**) (Nurse vs HCA **0.01**)
Suggesting	GP (n=96)	0.29±0.71	3.17±0.88	3.24±0.80	7.82±3.28	4.14±0.63	2.38±0.92	3.51±0.86	4.06±0.65	3.75±0.83
	Nurse (n=48)	1.10±2.46	3.54±0.78	3.58±0.88	8.56±3.12	3.93±1.10	2.84±1.04	3.35±0.90	4.24±0.60	4.13±0.66
	HCA (n=21)	0.14±0.48	3.15±0.71	3.55±0.82	5.14±5.04	3.73±0.88	3.27±1.03	3.21±0.70	3.87±0.52	3.62±0.65
	p	0.07	**0.02** (GP vs Nurse **0.01**)	0.81	**<0.001** (Nurse vs HCA **<0.001**)	0.25	**<0.001** (GP vs Nurse **0.01**) (GP vs HCA **<0.001**)	0.21	0.09	**0.01** (GP vs Nurse **0.01**)

Past behaviour and proximal goals were 10-point scales, i.e. for how many of the last 10 patients did the clinician perform the behaviour ('past behaviour'), and for how many of their next 10 patients does the clinician plan to perform the behaviour ('proximal goals'); the other measures were 5-point Likert scales: 1-strongly disagree', '2-disagree', '3-neither agree or disagree', '4-agree', and '5-strongly agree'.
Data presented as mean ± standard deviation.
p = test of differences between professional groups determined using Kruskal-Wallis (top level p value), with results of post-hoc Mann Whitney tests (with adjustment of critical value of p) presented in parentheses. GP, general practitioner; HCA, healthcare assistant; SCT, social cognitive theory; NPT, normalisation process theory. Statistically significant differences are indicated in bold font. Note: post-hoc analysis for communal specification (informing) did not identify statistically significant differences between groups following adjustment of critical value of p.

For peer review only - http://bmjopen.bmj.com/site/about/guidelines.xhtml

The SCT predictors for informing accounted for a small amount of variance (Cox & Snell R^2 0.05; Nagelkerke R^2 0.09) (Table 4). The covariate background information/no background information (OR=2.81; p=0.03) was statistically significant, indicating that it does appear to be associated with responses about informing patients about the links. Neither self-efficacy (OR=1.07, p=0.82), outcome expectations (OR=1.49, p=0.21) or proximal goals (OR=1.10, p=0.21) were significant predictors in a model that controlled for demographic factors and included other SCT constructs.

Behaviour 2: Suggesting patients with poorly controlled diabetes go for a dental check-up

All three professional groups reported suggesting patients with poorly controlled diabetes go for a dental check-up to none or one of the last 10 of their patients, with HCAs reporting the least amount (0.14±0.48), followed by GPs (0.29±0.71) and then nurses (1.10±2.46) (Table 3). The GPs' mean self-efficacy score (3.17±0.88) did not differ from that of HCAs (3.15±0.71), but was significantly lower than that of nurses (3.54±0.78) (p=0.01). The barriers that undermined self-efficacy to suggest patients go for a check-up most included 'it is not a priority for me/patient' and 'I am not set up for it', whereas the highest ranked item by all three professional groups was 'work being busy', with healthcare professionals scoring positively that they would remain confident to suggest patients with poorly controlled diabetes go for a check-up despite this challenge. GPs' mean score (3.24±0.80) for outcome expectations did not differ from that of HCAs (3.55±0.82) or nurses (3.58±0.88). For proximal goals (intention to suggest to go for a check-up), the responses were generally positive with the GPs' mean score (7.82±3.28) not differing from that of nurses (8.56±3.12), but the nurses' mean score was significantly higher than that of HCAs (5.14±5.04) (p<0.001).

The NPT responses for differentiation indicated that this behaviour was considered different from usual practice with GPs responding most strongly (4.14±0.63), but not significantly different to nurses (3.93±1.10) and HCAs (3.73±0.88). Whilst the GPs' mean response score to 'staff in the organisation share understanding of the purpose of suggesting' (2.38±0.92) was statistically significantly lower than that of nurses (2.84±1.04) (p=0.01) and HCAs (3.27±1.03) (p<0.001), for individual specification, there was no significant difference detected between GPs (3.51±0.86), nurses (3.35±0.90) and HCAs (3.21±0.70). Responses for internalisation and legitimation were positive. Nurses' mean score (4.24±0.60) did not differ from that of GPs (4.06±0.65) or HCAs (3.87±0.52) for seeing the potential value of suggesting to go for a dental check-up. For legitimation, nurses' mean score (4.13±0.66) did

For peer review only - http://bmjopen.bmj.com/site/about/guidelines.xhtml

not differ from that of HCAs (3.62±0.65), but was significantly higher than that of GPs (3.75±0.83) (p=0.01) for believing that suggesting patients go for a dental check-up was a legitimate part of their role.

The SCT predictors for suggesting patients go for a dental check-up accounted for a small amount of variance (Cox & Snell R^2 0.04; Nagelkerke R^2 0.07) (Table 4). The covariate background information/no background information (OR=1.26; p=0.57) was not associated with suggesting to go for a check-up, and outcome expectations (OR=1.06, p=0.81) and proximal goals (OR=0.99, p=0.90) were not statistically significant. Self-efficacy accounted for a statistically significant amount of variability (OR=1.71, p=0.04).

For peer review only

For peer review only - http://bmjopen.bmj.com/site/about/guidelines.xhtml

Table 4 Multivariate logistic regression model predicting past informing and suggesting (n=165)

Behaviours	Covariates and SCT Predictors	OR	SE	p	95% CI (B Coefficient)	
					Lower	Upper
Informing[1]	Background/no background	2.81	0.47	**0.03**	1.11	7.10
	Self-efficacy	1.07	0.30	0.82	0.60	1.92
	Outcome expectations	1.49	0.32	0.21	0.80	2.79
	Proximal goals	1.10	0.07	0.21	0.95	1.27
Suggesting[2]	Background/no background	1.26	0.40	0.57	0.58	2.74
	Self-efficacy	1.71	0.27	**0.04**	1.02	2.88
	Outcome expectations	1.06	0.26	0.81	0.64	1.75
	Proximal goals	0.99	0.06	0.90	0.88	1.12

p: statistically significant predictors indicated in bold font. OR, odds ratio; SE, standard error; CI confidence interval; B, exponential of β (odds ratio).

[1]Cox & Snell R^2 0.05, Nagelkerke R^2 0.09

[2]Cox & Snell R^2 0.04, Nagelkerke R^2 0.07

For peer review only

DISCUSSION

An abundance of evidence links periodontitis and diabetes, including multiple meta-analyses and two Cochrane reviews which have confirmed the benefits of periodontitis treatment on diabetes control. Adding to a number of recommendation documents for clinical practice that have been published over the last decade or so, the European Federation of Periodontology (EFP) and International Diabetes Federation (IDF) held a joint workshop in 2017 and produced outputs which were published simultaneously in the Journal of Clinical Periodontology and Diabetes Research and Clinical Practice,[40, 41] including guidelines for healthcare professionals, dental professionals and patients on the management of diabetes and periodontitis. Despite the existence of evidence and recommendations, the present research confirms that key clinical actions involved in enacting these recommendations are under-performed. The present research also identified modifiable correlates associated with variation in reported performance, suggesting targets for intervention to improve guideline recommendation uptake. Our cross sectional survey investigated the quantitative self-reports of GPs, nurses and HCAs for two extant best practice clinical behaviours in relation to diabetes and periodontitis care published at the time of our research.[16, 17] These included informing patients about the links between periodontitis and diabetes and suggesting patients with poorly controlled diabetes go for a dental check-up.

Whilst SCT and NPT have previously been used effectively in health promotion in the context of chronic disease management,[42-44] the combination of these behavioural (SCT) and implementation (NPT) theories represents a novel approach to investigate clinicians' practices and behavioural correlates. We have previously reported on the practices of dental clinicians in relation to the management of patients with diabetes and periodontitis, again using a combination of SCT and NPT.[34] We identified that according to self-reports, dental professionals are highly likely to inform their patients about the links between the two diseases and consider the impact of periodontitis treatment on glycaemic control, but there was very little evidence of dental clinicians contacting the patient's GP, with the respondents indicating that they would tend to communicate with the GP via the patient, as opposed to using direct communication or referral mechanisms. Based on the self-reported medical healthcare professionals' responses in this study, there was little to no informing patients about the links between diabetes and periodontitis or suggesting that patients go for a dental check-up. All professional groups agreed that although both behaviours differed from usual

For peer review only - http://bmjopen.bmj.com/site/about/guidelines.xhtml

ways of working (differentiation), there was potential value in the behaviours, indicated through positive responses for internalisation and intention (proximal goals).

For both of the investigated behaviours, nurses' responses were significantly higher than those of GPs and HCAs for self-efficacy; and for proximal goals, GPs' and nurses' scores were statistically significantly higher than those of HCAs. Regarding legitimation, nurses scored statistically significantly higher than GPs for informing patients about the links; and significantly higher than GPs and HCAs for suggesting patients go for a dental check-up. Perhaps these responses reflect the pivotal role that a nurse often plays in patient education and health promotion in diabetes management.[45, 46]

Diabetes management can be complex and it is therefore no surprise that 'it is not a priority for me/patient' should be ranked as having the potential to undermine self-efficacy for both behaviours within this context, however problems associated with accessing NHS dental care and work being busy would not prevent the respondents from performing the behaviours. Previous research identified barriers to healthcare professionals talking to patients with diabetes about the oral complications of diabetes,[19, 47] but the present study would appear to indicate some investment potential which has not been reported in the literature before and which could improve the implementation of guidance. The low levels of current behaviours may explain the small amount of variance seen with the SCT predictors. Notwithstanding, background information, whilst not a statistically significant predictor for suggesting to go for a check-up, was statistically significant for informing; and self-efficacy was a significant predictor for suggesting, and these should be considered as targets for training and implementation interventions. Future research could design an intervention to target these factors, which is then evaluated in a cluster-randomized trial to assess whether the intervention increased the target behaviours.

CONCLUSION

Despite strong evidence and best practice recommendations, healthcare professionals report low levels of informing patients with diabetes about the links between diabetes and periodontitis and suggesting to go for a dental check-up. However, healthcare professionals, particularly nurses, value these behaviours and perceive them to be legitimate to their role as diabetes educators. Professional role appears to be an important factor when considering who, in the diabetes multidisciplinary team, is best aligned to speak to patients about diabetes and periodontitis.

For peer review only - http://bmjopen.bmj.com/site/about/guidelines.xhtml

Acknowledgements. We thank the participants who generously gave their time and the North East and North Cumbria (NENC) and South West Peninsula (SWP) Clinical Research Networks for facilitating recruitment.

Competing interests. None declared.

Funding. This research was funded by a UK National Institute for Health Research Doctoral Research Fellowship (DRF-2014-07-023) awarded to SMB.

Contribution statement.

SMB, TR, PMP and JP contributed to the conception and design of the study. SMB collected the data, which was supervised by TR, PMP and JP. JP, TR and PMP were major contributors in analysis and interpretation of the data and in writing the manuscript. SMB, TR, PMP and JP were involved in drafting the work, revising it critically for important intellectual content and the final approval of the version published. SMB, TR, PMP and JP agree to be accountable for all aspects of the work. Members of Newcastle School of Dental Sciences Patient and Public Involvment group contributed to the study design and development.

Data sharing statement. A PDF of the online survey has been made available as a supplementary file. Permission is required from the authors for re-use.

For peer review only

REFERENCES

1 Diabetes UK. Number of people with diabetes in UK. Available at: https://www.diabetes.org.uk/about_us/news/new-stats-people-living-with-diabetes/ Accessed 03/05/2019, 2019.

2 P.M. Preshaw, A.L. Alba, D. Herrera, *et al.* Periodontitis and diabetes: a two-way relationship, Diabetol 55 (2012) 21-31.

3 N.J. Kassebaum, E. Bernabé, M. Dahiya, *et al.* Global burden of severe periodontitis in 1990-2010: a systematic review and meta-regression, J Dent Res 93 (2014) 1045-53.

4 D. White, N. Pitts, J.G. Steele, *et al.* Diseases and related disorders. London: NHS Information Centre for Health and Social Care, 2011.

5 H. Löe. Periodontal disease: the sixth complication of diabetes mellitus, Diabetes Care 16 (1993) 329-34.

6 B.L. Mealey, G.L. Ocampo. Diabetes mellitus and periodontal disease, Periodontol 2000 44 (2007) 127-53.

7 C. Tsai, C. Hayes, G.W. Taylor. Glycemic control of type 2 diabetes and severe periodontal disease in the US adult population, Community Dent Oral Epidemiol 30 (2002) 182-92.

8 B.L. Pihlstrom, B.S. Michalowicz, N.W. Johnson. Periodontal diseases, Lancet 366 (2005) 1809-20.

9 W.A. Soskolne, A. Klinger. The relationship between periodontal diseases and diabetes: an overview, Ann Periodontol 6 (2001) 91-8.

10 D. Polak, L. Shapira. An update on the evidence for pathogenic mechanisms that may link periodontitis and diabetes, J Clin Periodontol 45 (2018) 150-66.

11 L. Darré, J.N. Vergnes, P. Gourdy, M. Sixou. Efficacy of periodontal treatment on glycaemic control in diabetic patients: a meta-analysis of interventional studies, Diabetes Metab 34 (2008) 497-506.

For peer review only

For peer review only - http://bmjopen.bmj.com/site/about/guidelines.xhtml

12 S.J. Janket, A. Wightman, A.E. Baird*, et al.* Does periodontal treatment improve glycemic control in diabetic patients? A meta-analysis of intervention studies, J Dent Res 84 (2005) 1154-59.

13 T.C. Simpson, I. Needleman, S.H. Wild*, et al.* Treatment of periodontal disease for glycaemic control in people with diabetes, Cochrane Database Syst Rev (2010) CD004714.

14 T.C. Simpson, J.C. Weldon, H.V. Worthington*, et al.* Treatment of periodontal disease for glycaemic control in people with diabetes mellitus, Cochrane Database Syst Rev (2015) CD004714.

15 I.M. Stratton, A.I. Adler, H.A. Neil*, et al.* Association of glycaemia with macrovascular and microvascular complications of type 2 diabetes (UKPDS 35): prospective observational study, BMJ 321 (2000) 405-12.

16 International Diabetes Federation 2009. IDF Oral health for people with diabetes: guidelines. Available at: https://www.idf.org/e-library/guidelines/83-oral-health-for-people-with-diabetes.html Accessed 03/06/2019.

17 I.L.C. Chapple, R. Genco. Diabetes and periodontal diseases: consensus report of the Joint EFP/AAP Workshop on Periodontitis and Systemic Diseases, J Clin Periodontol 40 (2013) S106-S12.

18 A.C. Larme, J.A. Pugh. Evidence-based guidelines meet the real world. The case of diabetes care, Diabetes Care 24 (2001) 1728-33.

19 S.M. Bissett, K.M. Stone, T. Rapley, P.M. Preshaw. An exploratory qualitative interview study about collaboration between medicine and dentistry in relation to diabetes management, BMJ Open 3 (2013) e002192.

20 A.D. von Elm E, Egger M, Pocock SJ, Gotzsche PC, Vandenbroucke JP. . The Strengthening the Reporting of Observational Studies in Epidemiology (STROBE) Statement: guidelines for reporting observational studies.,

21 A. Bandura. Self-efficacy: the Exercise of Control. New York: Freeman, 1997.

22 M. Campbell, R. Fitzpatrick, A. Haines*, et al.* Framework for design and evaluation of complex interventions to improve health, BMJ 321 (2000) 694-96.

For peer review only - http://bmjopen.bmj.com/site/about/guidelines.xhtml

23 A. Bandura. Self-efficacy: toward a unifying theory of behavioral change, Psychol Rev 84 (1977) 191-215.

24 A. Bandura. Social Foundations of Thought and Action: a Social Cognitive Theory. New Jersey, USA: Englewood Cliffs, 1986.

25 C. May, T. Rapley, F. Mair*, et al.* Normalization process theory on-line users' manual, toolkit and NoMAD instrument. Available at: http://www.normalizationprocess.org Accessed 03/06/2019, 2015.

26 T.L. Finch, M. Girling, C.R. May*, et al.* Nomad: implemetation measure based on normalization process theory. [Measurement instrument]. Available at: http://www.normalizationprocess.org Accessed 03/06/2019, 2015.

27 G. Godin, A. Bélanger-Gravel, M. Eccles, J. Grimshaw. Healthcare professionals' intentions and behaviours: A systematic review of studies based on social cognitive theories, Implement Sci 3 (2008) 36.

28 J. Presseau, M. Johnston, J. Francis*, et al.* Theory-based predictors of multiple clinician behaviors in the management of diabetes, J Behav Med 37 (2014) 607-20.

29 F.G. Stacey, E.L. James, K. Chapman*, et al.* A systematic review and meta-analysis of social cognitive theory-based physical activity and/or nutrition behavior change interventions for cancer survivors, J Cancer Surviv 9 (2015) 305-38.

30 C. May, T. Finch, F. Mair*, et al.* Understanding the implementation of complex interventions in health care: the normalization process model, BMC Health Serv. Res. 7 (2007) 148.

31 C. May, T. Finch. Implementation, embedding, and integration: an outline of normalization process theory, Sociology 43 (2009) 535-54.

32 T. Rapley, M. Girling, F.S. Mair*, et al.* Improving the normalization of complex interventions: part 1 - development of the NoMAD instrument for assessing implementation work based on normalization process theory (NPT), BMC Med Res Methodol 18 (2018) 133-33.

For peer review only - http://bmjopen.bmj.com/site/about/guidelines.xhtml

33 T.L. Finch, M. Girling, C.R. May, *et al.* Improving the normalization of complex interventions: part 2 - validation of the NoMAD instrument for assessing implementation work based on normalization process theory (NPT), BMC Med Res Methodol 18 (2018) 135.

34 S.M. Bissett, J. Presseau, T. Rapley, P.M. Preshaw. Uptake of best practice recommendations in the management of patients with diabetes and periodontitis: a cross-sectional survey of dental clinicians, BDJ 226 (2019) 131-37.

35 M. Eccles, S. Hrisos, J. Francis, *et al.* Instrument development, data collection, and characteristics of practices, staff, and measures in the Improving Quality of Care in Diabetes (iQuaD) Study, Implement Sci 6 (2011) 61.

36 L. Clark, S. Ronaldson, L. Dyson, *et al.* Electronic prompts significantly increase response rates to postal questionnaires: a randomized trial within a randomized trial and meta-analysis, J Clin Epidemiol 68 (2015) 1446-50.

37 A. Rashidian, J. Miles, D. Russell, I. Russell. Sample size for regression analyses of theory of planned behaviour studies: Case of prescribing in general practice, Brit J Health Psych 11 (2006) 581-93.

38 J. Cohen. A power primer, Psychol Bull 112 (1992) 155-9.

39 L.J. Cronbach. Coefficient alpha and the internal structure of tests, Psychometrika 16 (1951) 297-334.

40 M. Sanz, A. Ceriello, M. Buysschaert, *et al.* Scientific evidence on the links between periodontal diseases and diabetes: consensus report and guidelines of the Joint EFP/IDF Workshop on Periodontal Diseases and Diabetes, Diabetes Res Clin Pract 137 (2018) 231-41.

41 M. Sanz, A. Ceriello, M. Buysschaert, *et al.* Scientific evidence on the links between periodontal diseases and diabetes: consensus report and guidelines of the Joint EFP/IDF Workshop on Periodontal Diseases and Diabetes, J Clin Periodontol 45 (2018) 138-49.

42 A. Bandura. Health promotion by social cognitive means, Health Educ Behav 31 (2004) 143-64.

For peer review only - http://bmjopen.bmj.com/site/about/guidelines.xhtml

43 A. Kennedy, P. Bower, D. Reeves, *et al.* Implementation of self management support for long term conditions in routine primary care settings: cluster randomised controlled trial, BMJ 346 (2013) f2882.

44 C.R. May, A. Cummings, M. Girling, *et al.* Using normalization process theory in feasibility studies and process evaluations of complex healthcare interventions: a systematic review, Implement Sci 13 (2018) 80.

45 L.M. Siminerio, M.M. Funnell, M. Peyrot, R.R. Rubin. US nurses' perceptions of their role in diabetes care, The Diabetes Educator 33 (2007) 152-62.

46 M. Peimani, O. Tabatabaei-Malazy, M. Pajouhi. Nurses' role in diabetes care: a review, J Diabetes Metab Disord 9 (2010) 4.

47 J. McDonald, R. Jayasuriya, M.F. Harris. The influence of power dynamics and trust on multidisciplinary collaboration: a qualitative case study of type 2 diabetes mellitus, BMC Health Serv. Res. 12 (2012) 63.

For peer review only

Block 1

Survey completion instructions.

The **purpose of this survey** is to assess the clinical practices of healthcare professionals with regard to their patients with **poorly controlled diabetes** (HbA1c > 58mmol/mol or > 7.5%). **All surveys are confidential.**
If you manage patients with diabetes, you are invited to complete this survey. Completion will take **around 30 minutes, for which you will be remunerated** at your professional rate.
The survey has 21 questions separated into two sections: section 1 asks **6 questions** about you; and section 2 **(15 questions)**, asks about the management of your patients with diabetes who are poorly controlled (HbA1c > 58mmol/mol or > 7.5%).
Please read each question carefully and answer it to the best of your ability. **There are no correct or incorrect responses**; we are merely interested in your point of view. The survey may appear to be repetitive since several of the statements are worded in a similar manner. It is the nature of this study that entails this methodological approach. We would **really appreciate your involvement**, as we wish to obtain **a 'snapshot' of current practice** in the UK regarding the dental management of people with diabetes.

Treating periodontitis (gum disease) can reduce HbA1c [4,5,6]

- Periodontitis impairs glycaemic control in people with diabetes1 and diabetes is a major risk factor for periodontitis (increasing the risk of periodontitis 3-fold).2
- A Cochrane systematic review reported a reduction in HbA1c of 4.4 mmol/mol (0.4 percentage points) 3-4 months after periodontal therapy,3 a clinical impact equivalent to that of many second-line pharmaceutical therapies for diabetes but without the risk of drug side-effects.

Aim of the research:

- The purpose of this survey is to find out the views and perspectives of healthcare professionals who manage patients with diabetes regarding the evidence supporting the links between diabetes and periodontitis.

References
1. Preshaw PM, Alba AL, Herrera D, Jepsen S, Konstantinidis A, Makrilakis K, et al. Periodontitis and diabetes: a two-way relationship. *Diabetologia* 2012;55:21-31.
2. Mealey BL, Oates TW, American Academy of P. Diabetes mellitus and periodontal diseases. *J Periodontol* 2006;77:1289-303.
3. Simpson TC, Needleman I, Wild SH, Moles DR, Mills EJ. Treatment of periodontal disease for glycaemic control in people with diabetes. *Cochrane Database Syst Rev* 2010:CD004714.
4. Borgnakke WS, Ylöstalo PV, Taylor GW, Genco RJ. Effect of periodontal disease on diabetes: systematic review of epidemiologic observational evidence. *J Clin Periodontol* 2013;40:S135-S52.
5. Janket SJ, Wightman A, Baird AE, Van Dyke TE, Jones JA. Does periodontal treatment improve glycemic control in diabetic patients? A meta-analysis of intervention studies. *J Dent Res* 2005;84:1154-59.
6. Darré L, Vergnes JN, Gourdy P, Sixou M. Efficacy of periodontal treatment on glycaemic control in diabetic patients: A meta-analysis of interventional studies. *Diabetes Metab* 2008;34:497-506.

Please click the arrows on the bottom right of the page to proceed.

Default Question Block

SECTION 1

Please answer these questions about yourself

Q1. Sex

Male ○ Female ○

Q2. Job

○ Community nurse
○ Diabetes nurse specialist
○ Diabetologist
○ Dietician
○ GP
○ GP with specialist interest in diabetes (with or without qualification)
○ GP with specialist interest in other area
○ Podiatrist
○ Practice nurse
○ Other (please specify)

Version 1.0

Q3. Age

- < 30
- 30 - 40
- 40 - 50
- 50 - 60
- > 60

Q4. Year of most recent professional qualification (yyyy - approximate is fine)?

Q5. How many patients with diabetes (either type 1 or 2) do you see in an average month?

Q6. Please use this space to clarify any responses or write any comments, if you would like to.

Block 2

SECTION 2

Please read each question carefully and answer it to the best of your ability.

There are no correct or incorrect responses; we are merely interested in your point of view.

Q7.

Thinking of **your last 10 patients with diabetes who you had a conversation with regarding their poor HbA1c levels (i.e. > 58 mmol/mol or > 7.5%).**

How many have you informed about the links between diabetes and periodontitis?

For example, if you inform everyone, then circle 10. If you inform some but not all, then chose a number which represents the proportion that you inform.

0 1 2 3 4 5 6 7 8 9 10

Q8.

Thinking of **your last 10 patients with diabetes who you had a conversation with regarding their poor HbA1c levels (i.e. > 58 mmol/mol or > 7.5%).**

How many have you ssuggested to go for a dental check-up?

For example, if you suggest it to everyone, then circle 10. If you inform some but not all, then chose a number which represents the proportion that you inform.

0 1 2 3 4 5 6 7 8 9 10

For peer review only - http://bmjopen.bmj.com/site/about/guidelines.xhtml

Version 1.0

Q9.
This question refers to **informing your patients with diabetes about the links between diabetes and periodontitis, during a conversation regarding their poor HbA1c levels (i.e. > 58 mmol/mol or > 7.5%).**

For each statement please **read the stem carefully first**, then select an answer that best suits your experience using the options.

For example, take a look at the stem and the first statement below and read them carefully. If you feel confident that you can inform your patient about the links between diabetes and periodontitis even when work is busy, then choose option 'Strongly Agree' or 'Agree'. If you feel that work being busy may affect your confidence in informing your patient about the links, then choose option 'Disagree' or 'Strongly Disagree'. If you are not sure, choose 'Neither agree nor disagree'. Now re-read the stem followed by the next statement, and so on.

Stem: I am confident that I can inform my patients with diabetes about the links between diabetes and periodontitis EVEN WHEN...

Statements:

	Strongly Disagree	Disagree	Neither Agree nor Disagree	Agree	Strongly Agree
... work is busy	○	○	○	○	○
... it is someone else's responsibility	○	○	○	○	○
... there is already too much to do	○	○	○	○	○
... it is not a priority for me	○	○	○	○	○
... it is not a priority for the patient	○	○	○	○	○
... I am not set up for it	○	○	○	○	○
... there are problems accessing dental services	○	○	○	○	○
... I am running late	○	○	○	○	○
... the patient has very few of their own teeth	○	○	○	○	○
... the patient seems to have good dental health	○	○	○	○	○
... my colleagues are doing the same	○	○	○	○	○
... my colleagues are not doing the same	○	○	○	○	○

Q10.
This question refers to **informing your patients with diabetes about the links between diabetes and periodontitis, during a conversation regarding their poor HbA1c levels (i.e. > 58 mmol/mol or > 7.5%).**

Stem: I am confident **that I can provide a suggestion to go for a dental check-up *EVEN* WHEN...**

Statements:

	Strongly Disagree	Disagree	Neither Agree nor Disagree	Agree	Strongly Agree
... work is busy	○	○	○	○	○
... it is someone else's responsibility	○	○	○	○	○
... there is already too much to do	○	○	○	○	○
... it is not a priority for me	○	○	○	○	○
... it is not a priority for the patient	○	○	○	○	○

... I am not set up for it	○	○	○	○	○
... there are problems accessing dental services	○	○	○	○	○
... I am running late	○	○	○	○	○
... the patient has very few of their own teeth	○	○	○	○	○
... the patient seems to have good dental health	○	○	○	○	○
... my colleagues are doing the same	○	○	○	○	○
... my colleagues are not doing the same	○	○	○	○	○

Q11.
This question is about **informing your <u>poorly controlled patients with diabetes</u> (HbA1c > 58 mmol/mol or > 7.5%) about the links between diabetes and periodontitis.**

Please read the statements carefully and chose an appropriate option.

OPTIONS

	Strongly agree	Agree	Neither Agree nor Disagree	Disagree	Strongly Disagree	Not relevant to my role	Not relevant at this stage	Not relevant to diabetes care
Statement 1: I can see how informing patients about the links between diabetes and periodontitis differs from my usual way of working	○	○	○	○	○	○	○	○
Statement 2: Staff in this organisation have a shared understanding of the purpose of informing patients about the links between diabetes and periodontitis	○	○	○	○	○	○	○	○
Statement 3: I understand how informing patients about the links between diabetes and periodontitis affects the nature of my own work	○	○	○	○	○	○	○	○
Statement 4: I can see the potential value of informing patients about the links between diabetes and periodontitis for my work	○	○	○	○	○	○	○	○
Statement 5: I believe that informing patients about the links between diabetes and periodontitis is a legitimate part of my role	○	○	○	○	○	○	○	○

Q12.
This question relates to **suggesting that your patients with HbA1c > 58 mmol/mol or > 7.5%, should go for a dental check-up.**

Please read the statements carefully and chose an appropriate option.

OPTIONS

	Strongly agree	Agree	Neither Agree nor Disagree	Disagree	Strongly Disagree	Not relevant to my role	Not relevant at this stage	Not relevant to diabetes care
Statement 1: I can see how suggesting that your patients with HbA1c > 58 mmol/mol or > 7.5%, should go for a dental check-up differs from my usual way of working	○	○	○	○	○	○	○	○
Statement 2: Staff in this organisation have a shared understanding of the purpose of suggesting that your patients with HbA1c > 58 mmol/mol or > 7.5%, should go for a dental check-up	○	○	○	○	○	○	○	○
Statement 3: I understand how suggesting that your patients with HbA1c > 58 mmol/mol or > 7.5%, should go for a dental check-up affects the nature of my own work	○	○	○	○	○	○	○	○
Statement 4: I can see the potential value of suggesting								

For peer review only - http://bmjopen.bmj.com/site/about/guidelines.xhtml

Version 1.0

that your patients with HbA1c > 58 mmol/mol or > 7.5%, should go for a dental check-up for my work	○	○	○	○	○	○	○	○
Statement 5: I believe that suggesting that your patients with HbA1c > 58 mmol/mol or > 7.5%, should go for a dental check-up is a legitimate part of my role	○	○	○	○	○	○	○	○

Q13.
Informing them about the links between diabetes and periodontitis will help my poorly controlled patients with diabetes (HbA1c > 58 mmol/mol or > 7.5%)?

Not at all	A little	Moderately	Quite a bit	Extremely
○	○	○	○	○

Q14.
Suggesting that they go for a dental check-up will help my patients with poorly controlled diabetes (HbA1c > 58 mmol/mol or > 7.5%)?

Not at all	A little	Moderately	Quite a bit	Extremely
○	○	○	○	○

Q15.
Iinforming my patients with poorly controlled diabetes (HbA1c > 58 mmol/mol or > 7.5%) about the links between diabetes and periodontitis, will be a good use of my time?

Never	Rarely	Sometimes	Often	All of the Time
○	○	○	○	○

Q16.
Suggesting that my patients with poorly controlled diabetes (HbA1c > 58 mmol/mol or >7.5%) go for a dental check-up will be a good use of my time?

Never	Rarely	Sometimes	Often	All of the Time
○	○	○	○	○

Q17.
This question asks about the **timing** of **informing** patients about the links between diabetes and periodontitis and **suggesting** to have a dental check-up. Please read the question and tick as many options as you like.

OPTIONS:

QUESTIONS:

	**Q1**: When is a good time to inform patients about the links between diabetes and periodontitis?	**Q2**: When is a good time to suggest having a dental check-up?
Never	☐	☐
At time of periodontal diagnosis	☐	☐
At time of diabetes diagnosis	☐	☐
At their routine dental check-up appointment	☐	☐
Alongside discussion regarding their HbA1c	☐	☐
When the patient has poorly controlled diabetes	☐	☐
When the patient is facing being prescribed oral medication (or additional oral medication) for their diabetes	☐	☐
When the patient is facing being put onto insulin or other injectables	☐	☐
At their first appointment in the practice	☐	☐
When the periodontal condition deteriorates	☐	☐
Other	☐	☐

Version 1.0

Q18.
This question asks about the **timing** of **informing** patients about the links between diabetes and periodontitis and **suggesting** to have a dental check-up. Please read the question and tick as many options as you like.

	OPTIONS - choose as many as you like							
	No one	GP	GP with special interest in diabetes	Dietician	Diabetologist	Dentist	Dental hygienist/therapist	Other
Q1: Who **could** inform about the links between diabetes and periodontitis?	☐	☐	☐	☐	☐	☐	☐	☐
Q2: Who **should** inform about the links between diabetes and periodontitis?	☐	☐	☐	☐	☐	☐	☐	☐
Q3: Who **could** suggest having a dental check-up?	☐	☐	☐	☐	☐	☐	☐	☐
Q4: Who **should** suggest having a dental check-up?	☐	☐	☐	☐	☐	☐	☐	☐

Q19.
This question relates to **your next 10 patients with poorly controlled diabetes (HbA1c>58 mmol/mol or > 7.5%).**

How many do you intend to inform about the links between diabetes and periodontitis?

0 1 2 3 4 5 6 7 8 9 10

Q20.
This question relates to **your next 10 patients with poorly controlled diabetes (HbA1c>58 mmol/mol or > 7.5%).**

How many do you intend to suggest going for a dental check-up?

0 1 2 3 4 5 6 7 8 9 10

Q21. Please use this space to clarify any responses or write any comments, if you would like to.

Reporting checklist for cross sectional study.

Based on the STROBE cross sectional guidelines.

Instructions to authors

Complete this checklist by entering the page numbers from your manuscript where readers will find each of the items listed below.

Your article may not currently address all the items on the checklist. Please modify your text to include the missing information. If you are certain that an item does not apply, please write "n/a" and provide a short explanation.

Upload your completed checklist as an extra file when you submit to a journal.

In your methods section, say that you used the STROBE cross sectionalreporting guidelines, and cite them as:

von Elm E, Altman DG, Egger M, Pocock SJ, Gotzsche PC, Vandenbroucke JP. The Strengthening the Reporting of Observational Studies in Epidemiology (STROBE) Statement: guidelines for reporting observational studies.

		Reporting Item	Page Number
**Title and abstract**			
Title	#1a	Indicate the study's design with a commonly used term in the title or the abstract	1

For peer review only

Abstract	#1b	Provide in the abstract an informative and balanced summary of what was done and what was found	2
**Introduction**			
Background / rationale	#2	Explain the scientific background and rationale for the investigation being reported	5
Objectives	#3	State specific objectives, including any prespecified hypotheses	6
**Methods**			
Study design	#4	Present key elements of study design early in the paper	7
Setting	#5	Describe the setting, locations, and relevant dates, including periods of recruitment, exposure, follow-up, and data collection	10
Eligibility criteria	#6a	Give the eligibility criteria, and the sources and methods of selection of participants.	10
	#7	Clearly define all outcomes, exposures, predictors, potential confounders, and effect modifiers. Give diagnostic criteria, if applicable	10
Data sources / measurement	#8	For each variable of interest give sources of data and details of methods of assessment (measurement). Describe comparability of assessment methods if there is more than one group. Give information separately for for exposed and unexposed groups if applicable.	10

For peer review only

Bias	#9	Describe any efforts to address potential sources of bias	4
Study size	#10	Explain how the study size was arrived at	11
Quantitative variables	#11	Explain how quantitative variables were handled in the analyses. If applicable, describe which groupings were chosen, and why	11
Statistical methods	#12a	Describe all statistical methods, including those used to control for confounding	11
Statistical methods	#12b	Describe any methods used to examine subgroups and interactions	11
Statistical methods	#12c	Explain how missing data were addressed	12
Statistical methods	#12d	If applicable, describe analytical methods taking account of sampling strategy	12
Statistical methods	#12e	Describe any sensitivity analyses	11
**Results**			
Participants	#13a	Report numbers of individuals at each stage of study—eg numbers potentially eligible, examined for eligibility, confirmed eligible, included in the study, completing follow-up, and analysed. Give information separately for for exposed and unexposed groups if applicable.	12
Participants	#13b	Give reasons for non-participation at each stage	12

For peer review only

Participants	#13c	Consider use of a flow diagram	
Descriptive data	#14a	Give characteristics of study participants (eg demographic, clinical, social) and information on exposures and potential confounders. Give information separately for exposed and unexposed groups if applicable.	13
Descriptive data	#14b	Indicate number of participants with missing data for each variable of interest	12
Outcome data	#15	Report numbers of outcome events or summary measures. Give information separately for exposed and unexposed groups if applicable.	15
Main results	#16a	Give unadjusted estimates and, if applicable, confounder-adjusted estimates and their precision (eg, 95% confidence interval). Make clear which confounders were adjusted for and why they were included	15
Main results	#16b	Report category boundaries when continuous variables were categorized	15
Main results	#16c	If relevant, consider translating estimates of relative risk into absolute risk for a meaningful time period	n/a
Other analyses	#17	Report other analyses done—e.g., analyses of subgroups and interactions, and sensitivity analyses	15
**Discussion**			
Key results	#18	Summarise key results with reference to study objectives	19

For peer review only

Limitations	#19	Discuss limitations of the study, taking into account sources of potential bias or imprecision. Discuss both direction and magnitude of any potential bias.	4
Interpretation	#20	Give a cautious overall interpretation considering objectives, limitations, multiplicity of analyses, results from similar studies, and other relevant evidence.	20
Generalisability	#21	Discuss the generalisability (external validity) of the study results	4
**Other Information**			
Funding	#22	Give the source of funding and the role of the funders for the present study and, if applicable, for the original study on which the present article is based	21

The STROBE checklist is distributed under the terms of the Creative Commons Attribution License CC-BY. This checklist was completed on 13. June 2019 using https://www.goodreports.org/, a tool made by the EQUATOR Network in collaboration with Penelope.ai

For peer review only

BMJ Open

Uptake of best practice recommendations in the management of patients with diabetes and periodontitis: a cross-sectional survey of healthcare professionals in primary care

Journal:	*BMJ Open*
Manuscript ID	bmjopen-2019-032369.R2
Article Type:	Original research
Date Submitted by the Author:	09-Dec-2019

Complete List of Authors:	Bissett, Susan; University of Newcastle upon Tyne, School of Dental Sciences<br>Rapley, Tim; Northumbria University Department of Social Work and Communities, Social Work, Education and Community Wellbeing<br>Preshaw, Philip; National University of Singapore, National University Centre for Oral Health<br>Presseau , Justin; Ottawa Hospital Research Institute,

**Primary Subject Heading**:	Diabetes and endocrinology
Secondary Subject Heading:	Dentistry and oral medicine
Keywords:	Diabetes, Periodontitis, Clinical behaviours, Best practice

SCHOLARONE™ Manuscripts

I, the Submitting Author has the right to grant and does grant on behalf of all authors of the Work (as defined in the below author licence), an exclusive licence and/or a non-exclusive licence for contributions from authors who are: i) UK Crown employees; ii) where BMJ has agreed a CC-BY licence shall apply, and/or iii) in accordance with the terms applicable for US Federal Government officers or employees acting as part of their official duties; on a worldwide, perpetual, irrevocable, royalty-free basis to BMJ Publishing Group Ltd ("BMJ") its licensees and where the relevant Journal is co-owned by BMJ to the co-owners of the Journal, to publish the Work in this journal and any other BMJ products and to exploit all rights, as set out in our licence.

The Submitting Author accepts and understands that any supply made under these terms is made by BMJ to the Submitting Author unless you are acting as an employee on behalf of your employer or a postgraduate student of an affiliated institution which is paying any applicable article publishing charge ("APC") for Open Access articles. Where the Submitting Author wishes to make the Work available on an Open Access basis (and intends to pay the relevant APC), the terms of reuse of such Open Access shall be governed by a Creative Commons licence – details of these licences and which Creative Commons licence will apply to this Work are set out in our licence referred to above.

Other than as permitted in any relevant BMJ Author's Self Archiving Policies, I confirm this Work has not been accepted for publication elsewhere, is not being considered for publication elsewhere and does not duplicate material already published. I confirm all authors consent to publication of this Work and authorise the granting of this licence.

For peer review only

Uptake of best practice recommendations in the management of patients with diabetes and periodontitis: a cross-sectional survey of healthcare professionals in primary care

Susan M Bissett,[1] Tim Rapley,[2] Philip M Preshaw,[1,3] Justin Presseau.[4,5]

*1 School of Dental Sciences, Newcastle University, Newcastle upon Tyne, UK

2 Faculty of Health and Life Sciences, Northumbria University, Newcastle upon Tyne, UK

3 National University Centre for Oral Health, National University of Singapore, Singapore

4 School of Epidemiology and Public Health, University of Ottawa, Ottawa, Canada

5 School of Psychology, University of Ottawa, Ottawa, Canada

Keywords: Diabetes; periodontitis; clinical behaviours; best practice

*Correspondence to: Dr Susan Bissett;

Email: s.m.bissett@newcastle.ac.uk

ORCiD ID (0000-0002-1997-3027)

For peer review only

ABSTRACT

Objectives: To investigate the practices of healthcare professionals in relation to best practice recommendations for the multidisciplinary management of people with diabetes and periodontitis, focusing on two clinical behaviours: informing patients about the links between diabetes and periodontitis, and suggesting patients with poorly controlled diabetes go for a dental check-up.

Design: Cross-sectional design utilising online questionnaires to assess self-reported performance and constructs from Social Cognitive Theory (SCT) and Normalisation Process Theory (NPT).

Setting: Primary care medical practices (n=37) in North East, North Cumbria and South West of England Clinical Research Networks.

Participants: 96 general practitioners (GPs), 48 nurses and 21 healthcare assistants (HCAs).

Results: Participants reported little to no informing patients about the links between diabetes and periodontitis or suggesting that they go for a dental check-up. Regarding future intent, both GPs (7.60±3.38) and nurses (7.94±3.69) scored significantly higher than HCAs (4.29±5.07) for SCT proximal goals (intention) in relation to informing patients about the links ($p<0.01$); and nurses (8.56±3.12) scored significantly higher than HCAs (5.14±5.04) for suggesting patients go for a dental check-up ($p<0.001$). All professional groups agreed on the potential value of both behaviours, and nurses scored significantly higher than GPs for legitimation (conforms to perception of job role) in relation to informing (nurses 4.16±0.71; GPs 3.77±0.76) and suggesting (nurses 4.13±0.66; GPs 3.75±0.83) (both $p<0.01$). The covariate background information (OR=2.81; $p=0.03$) was statistically significant for informing patients about the links.

Conclusions: Despite evidence-informed best practice recommendations, healthcare professionals currently report low levels of informing patients with diabetes about the links between diabetes and periodontitis and suggesting patients go for a dental check-up. However, healthcare professionals, particularly nurses, value these behaviours and consider them appropriate to their role. Whilst knowledge of the evidence is important, future

For peer review only - http://bmjopen.bmj.com/site/about/guidelines.xhtml

guidelines should consider different strategies to enable implementation of the delivery of healthcare interventions.

For peer review only

For peer review only - http://bmjopen.bmj.com/site/about/guidelines.xhtml

STRENGTHS AND LIMITATIONS OF THIS STUDY

- Although best practice recommendations for the multidisciplinary management of patients with diabetes and periodontitis have been published, there is limited research on their uptake; this is the first study to explore practices of UK medical healthcare professionals in the context of such recommendations.
- We achieved a high participation rate (80% at the practice level, and 76% at the participant level) from a range of healthcare professionals (general practitioners, nurses and healthcare assistants).
- It is acknowledged that generalizability may be limited given that participants were recuited by convenience sampling from two geographically separate locations in the UK (the North East and North Cumbria and South West Peninsula Clinical Research Networks) and the breakdown of GPs, nurses and HCAs who were sent the survey is unknown which means we cannot identify percentage response rate relative to job role.
- We utilised a novel combination of Social Cognitive Theory and Normalisation Process Theory to evaluate current practice at an individual and organisational level.
- The cross-sectional design precludes causal inference and direction of effect, but provides the first indication of factors associated with multidisciplinary management of people with diabetes and periodontitis in UK primary medical care.

For peer review only

For peer review only - http://bmjopen.bmj.com/site/about/guidelines.xhtml

INRODUCTION

Diabetes is a prevalent chronic non-communicable disease that has significant impacts on well-being and quality of life. Type 1 and type 2 diabetes are the principal categories, and in the UK as of 2019, there were 3.8 million people diagnosed with diabetes, of whom around 90% have type 2 diabetes. In addition, almost 1 million people are thought to be living with type 2 diabetes that is not yet diagnosed. This brings the estimated total number of people affected by diabetes to 4.7 million, or approximately one in 10 of the UK population over 40 years of age. This figure is predicted to reach 5.5 million by 2030.[1] Treatment of diabetes requires a life-long management strategy and varies in complexity given the multifactorial aetiology of the condition.

Periodontitis is also a prevalent chronic non-communicable disease. It is characterised by bacterially-driven inflammation in the tooth-supporting tissues that results in connective tissue destruction and alveolar bone resorption, leading, ultimately to tooth loss.[2] The dental plaque biofilm initiates and perpetuates the inflammation and an early sign of gingival inflammation (gingivitis) is bleeding, for example after brushing the teeth. Periodontitis is generally slowly progressing and painless, thus patients commonly present with advanced periodontitis that they were hitherto unaware of or ignored, even if they had been aware of some bleeding. Severe periodontitis has been reported to be the sixth-most prevalent disease in the world[3] and prevalence data in the UK have shown 8% of adults have advanced periodontitis.[4]

Periodontitis was initially identified as being a complication of diabetes in the early 1990s, the risk of periodontitis being increased by 2-3 times in a person with poorly controlled diabetes compared to individuals without.[5, 6] The pathogenic mechanisms linking periodontitis and diabetes are not yet fully understood but likely relate to upregulated systemic inflammation in each condition adversely affecting the other. The level of glycaemic control is key in determining risk,[7] and similar to the other complications of diabetes, the risk for periodontitis increases with poorer glycaemic control.[8-10]

Evidence supporting the potential to improve glycaemic control by treating periodontitis has emerged in more recent years, with several meta-analyses and two Cochrane reviews confirming reductions in HbA1c following effective periodontal therapy of up to 3-4 mmol/mol (0.3-0.4%) 3-4 months after periodontal treatment.[11-14] The precise mechanisms for the reduction of HbA1c following periodontal treatment are not completely

For peer review only - http://bmjopen.bmj.com/site/about/guidelines.xhtml

clear but likely arise from the combined effects of reduced inflammation and decreased bacterial challenge systemically, leading to a reduction in the systemic inflammatory state, and improvements in insulin resistance and insulin signalling.[10] Any reduction in HbA1c is important in people with diabetes, as it reduces the risk of diabetic complications. Every 1% reduction in HbA1c is associated with 21% reduced risk of any endpoint related to diabetes, 21% for deaths related to diabetes, 14% for myocardial infarction and 37% for microvascular complications.[15]

Over the years, a number of working groups have been established to provide guidance and recommendations for the multidisciplinary management of patients with periodontitis and diabetes. In 2007, the World Dental Federation (WDF) and the International Diabetes Federation (IDF) jointly organised a symposium on Oral Health and Diabetes. Experts agreed that there was urgent need to inform professionals, people with diabetes, policy makers and the public about the impact of diabetes on oral health; and they produced the IDF Guideline on Oral Health for People with Diabetes.[16] This guideline recommends that healthcare professionals should advise people with diabetes that good oral hygiene and regular dental checks are important and, in addition, adequate oral hygiene should be considered a normal part of diabetes self-management. In 2012, experts from the European Federation of Periodontology (EFP) and American Academy of Periodontology (AAP) also reviewed the evidence regarding the associations between diabetes and periodontitis.[17] They concluded that periodontitis was an independent predictor of several systemic conditions, including diabetes, and should be acknowledged as a major public health issue. Their manifesto stated that dental and medical communities should unite to develop a multidisciplinary approach to patient care.

Notwithstanding, previous research has shown that the academic and organisational silos in which dental and medical healthcare teams operate would appear to hinder shared knowledge and effective joint management of the two conditions.[18, 19] As knowledge alone is not sufficient to enable implementation of the delivery of healthcare interventions, research to identify the investment potential of healthcare professionals and enablers of change to aid implementation would be valued.

Accordingly, this study investigated the reported practices of healthcare professionals in relation to the management of diabetes and periodontitis to ascertain whether published best practice recommendations[16, 17] were being followed and to assess the factors which

For peer review only - http://bmjopen.bmj.com/site/about/guidelines.xhtml

predict behaviour, focusing on two recommended clinical behaviours: 1) Informing patients with diabetes about the links between diabetes and periodontitis; and 2) suggesting patients with poorly controlled diabetes go for a dental check-up.

METHODS

Design

The study used a cross-sectional design, involving online questionnaires (Qualtrics) to collect healthcare professionals' self-reported performance and views on the two clinical behaviours. The STROBE cross sectional reporting guidelines were followed.[20] As knowledge (or lack of knowledge) can influence cognitions towards behaviours, creating response bias,[21] we randomised (1:1 ratio) the provision (or not) of background information on the topic of the bidirectional relationship between diabetes and periodontitis as a preface at the start of the questionnaire, and the questionnaire was also piloted prior to use with healthcare professionals. As recommended by the UK Medical Research Council guidance for developing and evaluating complex interventions,[22] theory was used to explore healthcare professionals' behaviours at an individual and organisational level in the context of diabetes and periodontitis, specifically a combination of Social Cognitive Theory (SCT)[23, 24] and Normalisation Process Theory (NPT).[25, 26] A sample of the survey is included as a supplementary file.

SCT is a theory of motivation and action that describes key modifiable cognitions that can help to explain and improve the quality of care.[27-29] SCT holds that the care provided by healthcare professionals is a function of their self-efficacy (belief in their ability to provide the care), their outcome expectations (beliefs about the consequences of the care they provide), their proximal goals (intention to provide the care) and the present socio-structural determinants (external social and structural factors that act as barriers and enablers to care provision). NPT is an implementation theory used to identify, conceptualise and evaluate the factors that promote or inhibit the introduction, implementation and embedding of processes (such as patient management) into normal care.[30, 31] Subsequently, the NoMAD instrument[32, 33] was developed as a tool for using NPT to quantitatively assess implementation determinants, comprising four core constructs: coherence, cognitive participation, collective action, reflexive monitoring[30] and sub-constructs or items. The

For peer review only

NoMAD tool can be customised by selecting sub-constructs as appropriate according to the study context (Table 1).[34]

For peer review only

For peer review only - http://bmjopen.bmj.com/site/about/guidelines.xhtml

Table 1 Definitions of Social Cognitive Theory (SCT) and Normalisation Process Theory (NPT) constructs utilised in this research

Social Cognitive Theory (SCT): a theory of motivation and action that is used to predict healthcare professionals' cognitions that may improve quality of care. SCT comprises 3 constructs:		
	Self-efficacy	The belief in one's ability to succeed in specific situations or accomplish a task.
	Outcome Expectations	One's expectations about the consequences of performing an action or behaviour.
	Proximal Goals	One's intention (i.e. motivation) that regulates future effort and action with respect to a particular behaviour.
Normalisation Process Theory (NPT): a framework that is used to evaluate the factors that promote or inhibit implementation of processes (such as specific aspects of patient management) into routine care. NPT comprises 4 core constructs:		
	Coherence	How healthcare professionals make sense of the behaviour or intervention, e.g. what it involves and why?
	Cognitive Participation	How healthcare professionals get involved and stay committed, e.g. can they see how they contribute?
	Collective Action	How healthcare professionals make it work in practice, e.g. what do they need to make it happen?
	Reflexive Monitoring	How healthcare professionals assess whether it is worth the effort, e.g. does it result in benefits to patient care?
NPT also includes up to 16 sub-constructs, and those that are relevant to the particular clinical scenario should be selected. We selected 5 NPT sub-constructs in this research, and the participants were asked to respond to these in the questionnaire:		
	Differentiation	I can see how the (behaviour) differs from usual ways of working.
	Communal specification	Staff in this organisation have a shared understanding of the purpose of this (behaviour).
	Individual specification	I understand how the (behaviour) affects the nature of my own work.
	Internalisation	I can see the potential value of the (behaviour) for my work.
	Legitimation	I believe that participating in the (behaviour) is a legitimate part of my role.

Table adapted from Bandura (SCT),[23, 24] May et al. and Finch et al. (NPT).[25, 26] Reproduced with permission from Bissett et al., British Dental Journal 2019;226:131-37.[34]

For peer review only - http://bmjopen.bmj.com/site/about/guidelines.xhtml

Measures

Self-reported past behaviour

The questionnaire measured past behaviour in relation to the last 10 patients with diabetes seen for whom the healthcare professionals reported performing any of the two recommended clinical behaviours (informing and suggesting). Response options ranged from 0 to 10 patients (i.e. the behaviour was performed on ‘x’ of their last 10 patients with diabetes) in order to simplify the estimation of that behaviour by the respondent, consistent with other studies that evaluated healthcare professionals’ provision of diabetes-related healthcare.[35]

SCT constructs.

SCT constructs (self-efficacy, outcome expectations and proximal goals) were assessed using multi-item scales for both of the recommended clinical behaviours. Proximal goals was assessed on a 10-point scale to directly estimate for how many of the respondents’ next 10 patients with diabetes they intended to engage in the behaviours. Self-efficacy and outcome expectations were also assessed for the two behaviours, using a 5-point Likert scale as follows: ‘1-strongly disagree’, ‘2-disagree’, ‘3-neither agree or disagree’, ‘4-agree’, and ‘5-strongly agree’. The wording of the items to assess SCT constructs was consistent with previous research.[35]

NPT constructs.

We customised the NoMAD tool to include five NPT sub-constructs: differentiation; communal specification; individual specification; internalisation; and legitimation.[31] All were measured using the same 5-point Likert scale as shown above. Multi-item questions were informed by our previous qualitative research in the context of the management of people with periodontitis and diabetes.[19]

Sample

Participants invited to complete the questionnaire included general practitioners (GPs), nurses and healthcare assistants (HCAs) working in primary care services involved in the care of people with diabetes. Participants were recruited via convenience sampling through the UK Clinical Research Network (CRN), specifically North East and North Cumbria (NENC) CRN and South West Peninsula (SWP) CRN. A network facilitator approached medical teams and gave them a study summary to consider. The number of practices that the CRN approached

For peer review only - http://bmjopen.bmj.com/site/about/guidelines.xhtml

was not provided. Expressions of interest were emailed to the researcher, who scheduled a telephone call to obtain a contact list of the staff members who managed people with diabetes. An email invitation containing a link to the questionnaire was then sent to each staff member. Respondents were given five weeks to complete and submit the questionnaire. During this time, two electronic reminders were sent as these have been shown to improve response:[36] one at three weeks following the initial invitation, and the other after four weeks, thus reminding the respondent that there was only one week left before the close of the survey. Completion and submission of the questionnaire was incentivised at half of the respondent's professional hourly salary, given the typical time to complete established during the pilot phase. The questionnaire responses were anonymous. An *a priori* sample size target of n=150 was set, consistent with thresholds suggested in systematic reviews of studies using constructs from behaviour theories to predict medical professional behaviour.[27, 37] The recruitment period ran from January to October 2016.

Statistical analysis

Statistical analysis was conducted using SPSS v23.0 for Windows. Summary sample characteristics and NPT data were calculated using descriptive analyses (means and standard deviations). The internal consistency of multi-item constructs was tested in order to combine results to a single mean score.[38, 39] Kruskal Wallis tests were used to identify significant differences in responses according to professional role (GPs, nurses, HCAs), with Mann Whitney post hoc tests with adjustment of the critical value of p as appropriate. SCT correlates of behaviour were assessed using binary univariate and multivariate logistic regression to identify construct predictors for each of the behaviours.

Ethics Approval

Approval was obtained from North West-Greater Manchester West NHS Research Ethics Committee (16/NW/0030). Site approval was granted from each practice principal who approached eligible staff members. Interested staff members were sent an email invitation to the survey and consented to participate by clicking the link and submitting the survey.

Patient and Public Involvement

Patient and public involvement (PPI) was integrated throughout the life-cycle of the study, from funding proposal through to dissemination of results. The PPI group (the Oral & Dental PPI group of Newcastle University's School of Dental Sciences) contributed to the study

For peer review only - http://bmjopen.bmj.com/site/about/guidelines.xhtml

design and development of the research question via meetings with the lead researcher, and there was PPI representation on the study steering committee which met at regular intervals during the research.

RESULTS

The contact details of 46 medical practices were forwarded to the researcher by CRN facilitators: 11 from NENC CRN and 35 from SWP CRN. Of these, a total of 37 practices took part in the study (80% practice-level participation rate): 10 from NENC (27%) and 27 from SWP (73%). 176 questionnaires were returned from 217 that were sent out. Reasons for non-participation and a breakdown of the job role of the 217 participants were not known. Partially completed questionnaires were deleted list-wise to achieve a final sample of 165: 96 GPs, 48 nurses and 21 HCAs (76% participant-level response rate). Sample sociodemographic and clinical practice descriptive statistics (Table 2) show that the mean (± standard deviation) number of patients with a diabetes diagnosis was 6.3%±2.4%, and 67.6% of practices operated a separate diabetes clinic. The majority of the respondents were female (72%). HCAs saw a greater mean number of patients with diabetes per month (37.7±40.8), compared to GPs (33.2±31.8) and nurses (29.7±26.0), although the standard deviations were large.

For peer review only - http://bmjopen.bmj.com/site/about/guidelines.xhtml

Table 2 Sample characteristics of study population (n=165)

Practice level characteristics (n=37)		
Practice recruitment (N, %)	NENC	10 (27%)
	SWP	27 (73%)
List size (Min-Max)		3,600-35,818
Location (N, %)	Urban	7 (18.9%)
	Rural	11 (29.7%)
	Mixed	19 (51.4%)
Practices with separate diabetes clinic (N, %)		25 (67.6%)
% patient list >65 years (Mean ± SD)		22.5% ± 6.4%
% patient list have diabetes diagnosis (Mean ± SD)		6.3% ± 2.4%
Sample level characteristics (n=165)		
Sex (N, %)	Female	119 (72.1%)
	Male	46 (27.9%)
Age cohort (N, %)	< 30 years	5 (3.0%)
	30-40 years	39 (23.6%)
	40-50 years	58 (35.2%)
	50-60 years	56 (33.9%)
	> 60 years	7 (4.2%)
N patients with diabetes seen per month	GP (n=96)	33.2 ± 31.8
	Nurse (n=48)	29.7 ± 26.0
	HCA (n=21)	37.7 ± 40.8

Data for continuous variables presented as mean ± standard deviation.
%, percentage; GP, general practitioner; HCA, healthcare assistant.

For peer review only

For peer review only - http://bmjopen.bmj.com/site/about/guidelines.xhtml

Behaviour 1: Informing patients with diabetes about the links between diabetes and periodontitis

The questionnaire identified that healthcare professionals from all professional groups reported informing less than one of their last 10 patients with diabetes about the links between diabetes and periodontitis with no significant differences seen between GPs (0.23±0.69), nurses (0.58±1.81) and HCAs (0.24±0.63) (Table 3). The GPs' mean self-efficacy score (2.82±0.76) did not differ from that of HCAs (2.94±0.82), but was significantly lower than that of nurses (3.19±0.76) (p=0.01). The barriers perceived to undermine self-efficacy to inform patients included 'it is not a priority for the patient' and 'I am running late', whereas the highest ranked self-efficacy undermining barrier by all three professional groups was 'there are problems accessing dental services', with healthcare professionals scoring positively that they would remain confident to inform patients despite this challenge. The GPs' mean score (3.10±0.74) for outcome expectations did not differ significantly from that of HCAs (3.38±0.74), but was significantly lower than that of nurses (3.54±0.90) (p=0.01). For proximal goals (intention to inform) the responses were generally positive with GPs' mean score (7.60±3.38) not differing significantly from that of nurses (7.94±3.69); however, they were both significantly higher than that of HCAs (4.29±5.07) (p=0.01).

The NPT responses from all professional groups for differentiation indicated that this behaviour differed from usual ways of working with the GPs' mean response (4.06±0.89) not differing significantly from that of nurses (3.91±1.00) or HCAs (3.67±1.05). GPs' mean response score to communal specification (staff in the organisation share understanding of the purpose of informing) (2.27±0.83) did not differ from that of nurses (2.63±1.09) or HCAs (2.86±1.10). There were no significant differences detected for individual specification between GPs (3.40±0.93), nurses (3.30±0.95) and HCAs (3.07±0.70). Responses for internalisation and legitimation were positive. For internalisation (or seeing the potential value of informing), GPs' mean score (4.08±0.66) did not differ from that of nurses (4.26±0.61) or HCAs (4.07±0.59). The last cognitive participation item, 'I believe informing patients is a legitimate part of my role' was responded to positively. HCAs were the least positive (3.57±0.65) and significantly different to nurses (4.16±0.71), who also scored significantly higher than GPs (3.77±0.76) (p=0.01).

For peer review only - http://bmjopen.bmj.com/site/about/guidelines.xhtml

Table 3 Descriptive statistics of the two behaviours for SCT and NPT

**Behaviour**	**Job role**	**Past behaviour**	**Self-efficacy**	**Outcome expectations**	**Proximal goals**	**Differentiation**	**Communal specification**	**Individual specification**	**Internalisation**	**Legitimation**
Informing	GP (n=96)	0.23±0.69	2.82±0.76	3.10±0.74	7.60±3.38	4.06±0.89	2.27±0.83	3.40±0.93	4.08±0.66	3.77±0.76
	Nurse (n=48)	0.58±1.81	3.19±0.76	3.54±0.90	7.94±3.69	3.91±1.00	2.63±1.09	3.30±0.95	4.26±0.61	4.16±0.71
	HCA (n=21)	0.24±0.63	2.94±0.82	3.38±0.74	4.29±5.07	3.67±1.05	2.86±1.10	3.07±0.70	4.07±0.59	3.57±0.65
	p	0.95	**0.04** (GP vs Nurse **0.01**)	**0.01** (GP vs Nurse **0.01**)	**0.01** (GP vs HCA **0.01**) (Nurse vs HCA **0.01**)	0.29	**0.03**	0.25	0.32	**0.01** (GP vs Nurse **0.01**) (Nurse vs HCA **0.01**)
Suggesting	GP (n=96)	0.29±0.71	3.17±0.88	3.24±0.80	7.82±3.28	4.14±0.63	2.38±0.92	3.51±0.86	4.06±0.65	3.75±0.83
	Nurse (n=48)	1.10±2.46	3.54±0.78	3.58±0.88	8.56±3.12	3.93±1.10	2.84±1.04	3.35±0.90	4.24±0.60	4.13±0.66
	HCA (n=21)	0.14±0.48	3.15±0.71	3.55±0.82	5.14±5.04	3.73±0.88	3.27±1.03	3.21±0.70	3.87±0.52	3.62±0.65
	p	0.07	**0.02** (GP vs Nurse **0.01**)	0.81	**<0.001** (Nurse vs HCA **<0.001**)	0.25	**<0.001** (GP vs Nurse **0.01**) (GP vs HCA **<0.001**)	0.21	0.09	**0.01** (GP vs Nurse **0.01**)

Past behaviour and proximal goals were 10-point scales, i.e. for how many of the last 10 patients did the clinician perform the behaviour ('past behaviour'), and for how many of their next 10 patients does the clinician plan to perform the behaviour ('proximal goals'); the other measures were 5-point Likert scales: 1-strongly disagree', '2-disagree', '3-neither agree or disagree', '4-agree', and '5-strongly agree'.
Data presented as mean ± standard deviation.
p = test of differences between professional groups determined using Kruskal-Wallis (top level p value), with results of post-hoc Mann Whitney tests (with adjustment of critical value of p) presented in parentheses. GP, general practitioner; HCA, healthcare assistant; SCT, social cognitive theory; NPT, normalisation process theory. Statistically significant differences are indicated in bold font. Note: post-hoc analysis for communal specification (informing) did not identify statistically significant differences between groups following adjustment of critical value of p.

For peer review only

The SCT predictors for informing accounted for a small amount of variance (Cox & Snell R^2 0.05; Nagelkerke R^2 0.09) (Table 4). The covariate background information/no background information (OR=2.81; p=0.03) was statistically significant, indicating that it does appear to be associated with responses about informing patients about the links. Neither self-efficacy (OR=1.07, p=0.82), outcome expectations (OR=1.49, p=0.21) or proximal goals (OR=1.10, p=0.21) were significant predictors in a model that controlled for demographic factors and included other SCT constructs.

Behaviour 2: Suggesting patients with poorly controlled diabetes go for a dental check-up

All three professional groups reported suggesting patients with poorly controlled diabetes go for a dental check-up to none or one of the last 10 of their patients, with HCAs reporting the least amount (0.14±0.48), followed by GPs (0.29±0.71) and then nurses (1.10±2.46) (Table 3). The GPs' mean self-efficacy score (3.17±0.88) did not differ from that of HCAs (3.15±0.71), but was significantly lower than that of nurses (3.54±0.78) (p=0.01). The barriers that undermined self-efficacy to suggest patients go for a check-up most included 'it is not a priority for me/patient' and 'I am not set up for it', whereas the highest ranked item by all three professional groups was 'work being busy', with healthcare professionals scoring positively that they would remain confident to suggest patients with poorly controlled diabetes go for a check-up despite this challenge. GPs' mean score (3.24±0.80) for outcome expectations did not differ from that of HCAs (3.55±0.82) or nurses (3.58±0.88). For proximal goals (intention to suggest to go for a check-up), the responses were generally positive with the GPs' mean score (7.82±3.28) not differing from that of nurses (8.56±3.12), but the nurses' mean score was significantly higher than that of HCAs (5.14±5.04) (p<0.001).

The NPT responses for differentiation indicated that this behaviour was considered different from usual practice with GPs responding most strongly (4.14±0.63), but not significantly different to nurses (3.93±1.10) and HCAs (3.73±0.88). Whilst the GPs' mean response score to 'staff in the organisation share understanding of the purpose of suggesting' (2.38±0.92) was statistically significantly lower than that of nurses (2.84±1.04) (p=0.01) and HCAs (3.27±1.03) (p<0.001), for individual specification, there was no significant difference detected between GPs (3.51±0.86), nurses (3.35±0.90) and HCAs (3.21±0.70). Responses for internalisation and legitimation were positive. Nurses' mean score (4.24±0.60) did not differ from that of GPs (4.06±0.65) or HCAs (3.87±0.52) for seeing the potential value of suggesting to go for a dental check-up. For legitimation, nurses' mean score (4.13±0.66) did

For peer review only - http://bmjopen.bmj.com/site/about/guidelines.xhtml

not differ from that of HCAs (3.62±0.65), but was significantly higher than that of GPs (3.75±0.83) (p=0.01) for believing that suggesting patients go for a dental check-up was a legitimate part of their role.

The SCT predictors for suggesting patients go for a dental check-up accounted for a small amount of variance (Cox & Snell R^2 0.04; Nagelkerke R^2 0.07) (Table 4). The covariate background information/no background information (OR=1.26; p=0.57) was not associated with suggesting to go for a check-up, and outcome expectations (OR=1.06, p=0.81) and proximal goals (OR=0.99, p=0.90) were not statistically significant. Self-efficacy accounted for a statistically significant amount of variability (OR=1.71, p=0.04).

For peer review only

For peer review only - http://bmjopen.bmj.com/site/about/guidelines.xhtml

Table 4 Multivariate logistic regression model predicting past informing and suggesting (n=165)

Behaviours	Covariates and SCT Predictors	OR	SE	p	95% CI (B Coefficient)	
					Lower	Upper
Informing[1]	Background/no background	2.81	0.47	**0.03**	1.11	7.10
	Self-efficacy	1.07	0.30	0.82	0.60	1.92
	Outcome expectations	1.49	0.32	0.21	0.80	2.79
	Proximal goals	1.10	0.07	0.21	0.95	1.27
Suggesting[2]	Background/no background	1.26	0.40	0.57	0.58	2.74
	Self-efficacy	1.71	0.27	**0.04**	1.02	2.88
	Outcome expectations	1.06	0.26	0.81	0.64	1.75
	Proximal goals	0.99	0.06	0.90	0.88	1.12

p: statistically significant predictors indicated in bold font. OR, odds ratio; SE, standard error; CI confidence interval; B, exponential of β (odds ratio).

[1]Cox & Snell R^2 0.05, Nagelkerke R^2 0.09

[2]Cox & Snell R^2 0.04, Nagelkerke R^2 0.07

For peer review only

DISCUSSION

An abundance of evidence links periodontitis and diabetes, including multiple meta-analyses and two Cochrane reviews which have confirmed the benefits of periodontitis treatment on diabetes control. Adding to a number of recommendation documents for clinical practice that have been published over the last decade or so, the European Federation of Periodontology (EFP) and International Diabetes Federation (IDF) held a joint workshop in 2017 and produced outputs which were published simultaneously in the Journal of Clinical Periodontology and Diabetes Research and Clinical Practice,[40, 41] including guidelines for healthcare professionals, dental professionals and patients on the management of diabetes and periodontitis. Despite the existence of evidence and recommendations, the present research confirms that key clinical actions involved in enacting these recommendations are under-performed. The present research also identified modifiable correlates associated with variation in reported performance, suggesting targets for intervention to improve guideline recommendation uptake. Our cross sectional survey investigated the quantitative self-reports of GPs, nurses and HCAs for two extant best practice clinical behaviours in relation to diabetes and periodontitis care published at the time of our research.[16, 17] These included informing patients about the links between periodontitis and diabetes and suggesting patients with poorly controlled diabetes go for a dental check-up.

Whilst SCT and NPT have previously been used effectively in health promotion in the context of chronic disease management,[42-44] the combination of these behavioural (SCT) and implementation (NPT) theories represents a novel approach to investigate clinicians' practices and behavioural correlates. We have previously reported on the practices of dental clinicians in relation to the management of patients with diabetes and periodontitis, again using a combination of SCT and NPT.[34] We identified that according to self-reports, dental professionals are highly likely to inform their patients about the links between the two diseases and consider the impact of periodontitis treatment on glycaemic control, but there was very little evidence of dental clinicians contacting the patient's GP, with the respondents indicating that they would tend to communicate with the GP via the patient, as opposed to using direct communication or referral mechanisms. Based on the self-reported medical healthcare professionals' responses in this study, there was little to no informing patients about the links between diabetes and periodontitis or suggesting that patients go for a dental check-up. All professional groups agreed that although both behaviours differed from usual

For peer review only - http://bmjopen.bmj.com/site/about/guidelines.xhtml

ways of working (differentiation), there was potential value in the behaviours, indicated through positive responses for internalisation and intention (proximal goals).

For both of the investigated behaviours, nurses' responses were significantly higher than those of GPs and HCAs for self-efficacy; and for proximal goals, GPs' and nurses' scores were statistically significantly higher than those of HCAs. Regarding legitimation, nurses scored statistically significantly higher than GPs for informing patients about the links; and significantly higher than GPs and HCAs for suggesting patients go for a dental check-up. Perhaps these responses reflect the pivotal role that a nurse often plays in patient education and health promotion in diabetes management.[45, 46]

Whilst we achieved a high participation rate (80% at the practice level, and 76% at the participant level) from a range of healthcare professionals, generalizability may be limited given that participants were recuited by convenience sampling. Furthermore, and the breakdown of GPs, nurses and HCAs who were sent the survey is unknown which means we cannot identify percentage response rate relative to job role.

Diabetes management can be complex and it is therefore no surprise that 'it is not a priority for me/patient' should be ranked as having the potential to undermine self-efficacy for both behaviours within this context, however problems associated with accessing NHS dental care and work being busy would not prevent the respondents from performing the behaviours. Previous research identified barriers to healthcare professionals talking to patients with diabetes about the oral complications of diabetes,[19, 47] but the present study would appear to indicate some investment potential which has not been reported in the literature before and which could improve the implementation of guidance. The low levels of current behaviours may explain the small amount of variance seen with the SCT predictors. Notwithstanding, background information, whilst not a statistically significant predictor for suggesting to go for a check-up, was statistically significant for informing; and self-efficacy was a significant predictor for suggesting, and these should be considered as targets for training and implementation interventions. Future research could design an intervention to target these factors, which is then evaluated in a cluster-randomized trial to assess whether the intervention increased the target behaviours.

CONCLUSION

For peer review only - http://bmjopen.bmj.com/site/about/guidelines.xhtml

Despite strong evidence and best practice recommendations, healthcare professionals report low levels of informing patients with diabetes about the links between diabetes and periodontitis and suggesting to go for a dental check-up. However, healthcare professionals, particularly nurses, value these behaviours and perceive them to be legitimate to their role as diabetes educators. Professional role appears to be an important factor when considering who, in the diabetes multidisciplinary team, is best aligned to speak to patients about diabetes and periodontitis.

For peer review only

For peer review only - http://bmjopen.bmj.com/site/about/guidelines.xhtml

Acknowledgements. We thank the participants who generously gave their time and the North East and North Cumbria (NENC) and South West Peninsula (SWP) Clinical Research Networks for facilitating recruitment.

Competing interests. None declared.

Funding. This research was funded by a UK National Institute for Health Research Doctoral Research Fellowship (DRF-2014-07-023) awarded to SMB.

Contribution statement. SMB, TR, PMP and JP contributed to the conception and design of the study. SMB collected the data, which was supervised by TR, PMP and JP. JP, TR and PMP were major contributors in analysis and interpretation of the data and in writing the manuscript. SMB, TR, PMP and JP were involved in drafting the work, revising it critically for important intellectual content and the final approval of the version published. SMB, TR, PMP and JP agree to be accountable for all aspects of the work. Members of Newcastle School of Dental Sciences Patient and Public Involvment group contributed to the study design and development.

Data sharing statement. No additional data are available.

For peer review only

REFERENCES

1 Diabetes UK. Number of people with diabetes in UK. Available at: https://www.diabetes.org.uk/about_us/news/new-stats-people-living-with-diabetes/ Accessed 03/05/2019, 2019.

2 P.M. Preshaw, A.L. Alba, D. Herrera*, et al.* Periodontitis and diabetes: a two-way relationship, Diabetol 55 (2012) 21-31.

3 N.J. Kassebaum, E. Bernabé, M. Dahiya*, et al.* Global burden of severe periodontitis in 1990-2010: a systematic review and meta-regression, J Dent Res 93 (2014) 1045-53.

4 D. White, N. Pitts, J.G. Steele*, et al.* Diseases and related disorders. London: NHS Information Centre for Health and Social Care, 2011.

5 H. Löe. Periodontal disease: the sixth complication of diabetes mellitus, Diabetes Care 16 (1993) 329-34.

6 B.L. Mealey, G.L. Ocampo. Diabetes mellitus and periodontal disease, Periodontol 2000 44 (2007) 127-53.

7 C. Tsai, C. Hayes, G.W. Taylor. Glycemic control of type 2 diabetes and severe periodontal disease in the US adult population, Community Dent Oral Epidemiol 30 (2002) 182-92.

8 B.L. Pihlstrom, B.S. Michalowicz, N.W. Johnson. Periodontal diseases, Lancet 366 (2005) 1809-20.

9 W.A. Soskolne, A. Klinger. The relationship between periodontal diseases and diabetes: an overview, Ann Periodontol 6 (2001) 91-8.

10 D. Polak, L. Shapira. An update on the evidence for pathogenic mechanisms that may link periodontitis and diabetes, J Clin Periodontol 45 (2018) 150-66.

11 L. Darré, J.N. Vergnes, P. Gourdy, M. Sixou. Efficacy of periodontal treatment on glycaemic control in diabetic patients: a meta-analysis of interventional studies, Diabetes Metab 34 (2008) 497-506.

For peer review only - http://bmjopen.bmj.com/site/about/guidelines.xhtml

12 S.J. Janket, A. Wightman, A.E. Baird*, et al.* Does periodontal treatment improve glycemic control in diabetic patients? A meta-analysis of intervention studies, J Dent Res 84 (2005) 1154-59.

13 T.C. Simpson, I. Needleman, S.H. Wild*, et al.* Treatment of periodontal disease for glycaemic control in people with diabetes, Cochrane Database Syst Rev (2010) CD004714.

14 T.C. Simpson, J.C. Weldon, H.V. Worthington*, et al.* Treatment of periodontal disease for glycaemic control in people with diabetes mellitus, Cochrane Database Syst Rev (2015) CD004714.

15 I.M. Stratton, A.I. Adler, H.A. Neil*, et al.* Association of glycaemia with macrovascular and microvascular complications of type 2 diabetes (UKPDS 35): prospective observational study, BMJ 321 (2000) 405-12.

16 International Diabetes Federation 2009. IDF Oral health for people with diabetes: guidelines. Available at: https://www.idf.org/e-library/guidelines/83-oral-health-for-people-with-diabetes.html Accessed 03/06/2019.

17 I.L.C. Chapple, R. Genco. Diabetes and periodontal diseases: consensus report of the Joint EFP/AAP Workshop on Periodontitis and Systemic Diseases, J Clin Periodontol 40 (2013) S106-S12.

18 A.C. Larme, J.A. Pugh. Evidence-based guidelines meet the real world. The case of diabetes care, Diabetes Care 24 (2001) 1728-33.

19 S.M. Bissett, K.M. Stone, T. Rapley, P.M. Preshaw. An exploratory qualitative interview study about collaboration between medicine and dentistry in relation to diabetes management, BMJ Open 3 (2013) e002192.

20 A.D. von Elm E, Egger M, Pocock SJ, Gotzsche PC, Vandenbroucke JP. . The Strengthening the Reporting of Observational Studies in Epidemiology (STROBE) Statement: guidelines for reporting observational studies.,

21 A. Bandura. Self-efficacy: the Exercise of Control. New York: Freeman, 1997.

22 M. Campbell, R. Fitzpatrick, A. Haines*, et al.* Framework for design and evaluation of complex interventions to improve health, BMJ 321 (2000) 694-96.

For peer review only - http://bmjopen.bmj.com/site/about/guidelines.xhtml

23 A. Bandura. Self-efficacy: toward a unifying theory of behavioral change, Psychol Rev 84 (1977) 191-215.

24 A. Bandura. Social Foundations of Thought and Action: a Social Cognitive Theory. New Jersey, USA: Englewood Cliffs, 1986.

25 C. May, T. Rapley, F. Mair, *et al.* Normalization process theory on-line users' manual, toolkit and NoMAD instrument. Available at: http://www.normalizationprocess.org Accessed 03/06/2019, 2015.

26 T.L. Finch, M. Girling, C.R. May, *et al.* Nomad: implemetation measure based on normalization process theory. [Measurement instrument]. Available at: http://www.normalizationprocess.org Accessed 03/06/2019, 2015.

27 G. Godin, A. Bélanger-Gravel, M. Eccles, J. Grimshaw. Healthcare professionals' intentions and behaviours: A systematic review of studies based on social cognitive theories, Implement Sci 3 (2008) 36.

28 J. Presseau, M. Johnston, J. Francis, *et al.* Theory-based predictors of multiple clinician behaviors in the management of diabetes, J Behav Med 37 (2014) 607-20.

29 F.G. Stacey, E.L. James, K. Chapman, *et al.* A systematic review and meta-analysis of social cognitive theory-based physical activity and/or nutrition behavior change interventions for cancer survivors, J Cancer Surviv 9 (2015) 305-38.

30 C. May, T. Finch, F. Mair, *et al.* Understanding the implementation of complex interventions in health care: the normalization process model, BMC Health Serv. Res. 7 (2007) 148.

31 C. May, T. Finch. Implementation, embedding, and integration: an outline of normalization process theory, Sociology 43 (2009) 535-54.

32 T. Rapley, M. Girling, F.S. Mair, *et al.* Improving the normalization of complex interventions: part 1 - development of the NoMAD instrument for assessing implementation work based on normalization process theory (NPT), BMC Med Res Methodol 18 (2018) 133-33.

For peer review only

33 T.L. Finch, M. Girling, C.R. May, *et al.* Improving the normalization of complex interventions: part 2 - validation of the NoMAD instrument for assessing implementation work based on normalization process theory (NPT), BMC Med Res Methodol 18 (2018) 135.

34 S.M. Bissett, J. Presseau, T. Rapley, P.M. Preshaw. Uptake of best practice recommendations in the management of patients with diabetes and periodontitis: a cross-sectional survey of dental clinicians, BDJ 226 (2019) 131-37.

35 M. Eccles, S. Hrisos, J. Francis, *et al.* Instrument development, data collection, and characteristics of practices, staff, and measures in the Improving Quality of Care in Diabetes (iQuaD) Study, Implement Sci 6 (2011) 61.

36 L. Clark, S. Ronaldson, L. Dyson, *et al.* Electronic prompts significantly increase response rates to postal questionnaires: a randomized trial within a randomized trial and meta-analysis, J Clin Epidemiol 68 (2015) 1446-50.

37 A. Rashidian, J. Miles, D. Russell, I. Russell. Sample size for regression analyses of theory of planned behaviour studies: Case of prescribing in general practice, Brit J Health Psych 11 (2006) 581-93.

38 J. Cohen. A power primer, Psychol Bull 112 (1992) 155-9.

39 L.J. Cronbach. Coefficient alpha and the internal structure of tests, Psychometrika 16 (1951) 297-334.

40 M. Sanz, A. Ceriello, M. Buysschaert, *et al.* Scientific evidence on the links between periodontal diseases and diabetes: consensus report and guidelines of the Joint EFP/IDF Workshop on Periodontal Diseases and Diabetes, Diabetes Res Clin Pract 137 (2018) 231-41.

41 M. Sanz, A. Ceriello, M. Buysschaert, *et al.* Scientific evidence on the links between periodontal diseases and diabetes: consensus report and guidelines of the Joint EFP/IDF Workshop on Periodontal Diseases and Diabetes, J Clin Periodontol 45 (2018) 138-49.

42 A. Bandura. Health promotion by social cognitive means, Health Educ Behav 31 (2004) 143-64.

For peer review only

For peer review only - http://bmjopen.bmj.com/site/about/guidelines.xhtml

43 A. Kennedy, P. Bower, D. Reeves, *et al.* Implementation of self management support for long term conditions in routine primary care settings: cluster randomised controlled trial, BMJ 346 (2013) f2882.

44 C.R. May, A. Cummings, M. Girling, *et al.* Using normalization process theory in feasibility studies and process evaluations of complex healthcare interventions: a systematic review, Implement Sci 13 (2018) 80.

45 L.M. Siminerio, M.M. Funnell, M. Peyrot, R.R. Rubin. US nurses' perceptions of their role in diabetes care, The Diabetes Educator 33 (2007) 152-62.

46 M. Peimani, O. Tabatabaei-Malazy, M. Pajouhi. Nurses' role in diabetes care: a review, J Diabetes Metab Disord 9 (2010) 4.

47 J. McDonald, R. Jayasuriya, M.F. Harris. The influence of power dynamics and trust on multidisciplinary collaboration: a qualitative case study of type 2 diabetes mellitus, BMC Health Serv. Res. 12 (2012) 63.

For peer review only

Block 1

Survey completion instructions.

The **purpose of this survey** is to assess the clinical practices of healthcare professionals with regard to their patients with **poorly controlled diabetes** (HbA1c > 58mmol/mol or > 7.5%). **All surveys are confidential.**

If you manage patients with diabetes, you are invited to complete this survey. Completion will take **around 30 minutes, for which you will be remunerated** at your professional rate.

The survey has 21 questions separated into two sections: section 1 asks **6 questions** about you; and section 2 **(15 questions)**, asks about the management of your patients with diabetes who are poorly controlled (HbA1c > 58mmol/mol or > 7.5%).

Please read each question carefully and answer it to the best of your ability. **There are no correct or incorrect responses**; we are merely interested in your point of view. The survey may appear to be repetitive since several of the statements are worded in a similar manner. It is the nature of this study that entails this methodological approach. We would **really appreciate your involvement**, as we wish to obtain **a 'snapshot' of current practice** in the UK regarding the dental management of people with diabetes.

Treating periodontitis (gum disease) can reduce HbA1c [4,5,6]

- Periodontitis impairs glycaemic control in people with diabetes1 and diabetes is a major risk factor for periodontitis (increasing the risk of periodontitis 3-fold).2

- A Cochrane systematic review reported a reduction in HbA1c of 4.4 mmol/mol (0.4 percentage points) 3-4 months after periodontal therapy,3 a clinical impact equivalent to that of many second-line pharmaceutical therapies for diabetes but without the risk of drug side-effects.

Aim of the research:

- The purpose of this survey is to find out the views and perspectives of healthcare professionals who manage patients with diabetes regarding the evidence supporting the links between diabetes and periodontitis.

References
1. Preshaw PM, Alba AL, Herrera D, Jepsen S, Konstantinidis A, Makrilakis K, et al. Periodontitis and diabetes: a two-way relationship. *Diabetologia* 2012;55:21-31.
2. Mealey BL, Oates TW, American Academy of P. Diabetes mellitus and periodontal diseases. *J Periodontol* 2006;77:1289-303.
3. Simpson TC, Needleman I, Wild SH, Moles DR, Mills EJ. Treatment of periodontal disease for glycaemic control in people with diabetes. *Cochrane Database Syst Rev* 2010:CD004714.
4. Borgnakke WS, Ylöstalo PV, Taylor GW, Genco RJ. Effect of periodontal disease on diabetes: systematic review of epidemiologic observational evidence. *J Clin Periodontol* 2013;40:S135-S52.
5. Janket SJ, Wightman A, Baird AE, Van Dyke TE, Jones JA. Does periodontal treatment improve glycemic control in diabetic patients? A meta-analysis of intervention studies. *J Dent Res* 2005;84:1154-59.
6. Darré L, Vergnes JN, Gourdy P, Sixou M. Efficacy of periodontal treatment on glycaemic control in diabetic patients: A meta-analysis of interventional studies. *Diabetes Metab* 2008;34:497-506.

Please click the arrows on the bottom right of the page to proceed.

Default Question Block

SECTION 1

Please answer these questions about yourself

Q1. Sex

○ Male ○ Female

Q2. Job

○ Community nurse
○ Diabetes nurse specialist
○ Diabetologist
○ Dietician
○ GP
○ GP with specialist interest in diabetes (with or without qualification)
○ GP with specialist interest in other area
○ Podiatrist
○ Practice nurse
○ Other (please specify)

Version 1.0

Q3. Age

- ○ < 30
- ○ 30 - 40
- ○ 40 - 50
- ○ 50 - 60
- ○ > 60

Q4. Year of most recent professional qualification (yyyy - approximate is fine)?

Q5. How many patients with diabetes (either type 1 or 2) do you see in an average month?

Q6. Please use this space to clarify any responses or write any comments, if you would like to.

Block 2

SECTION 2

Please read each question carefully and answer it to the best of your ability.
There are no correct or incorrect responses; we are merely interested in your point of view.

Q7.
Thinking of **your last 10 patients with diabetes who you had a conversation with regarding their poor HbA1c levels (i.e. > 58 mmol/mol or > 7.5%).**

How many have you informed about the links between diabetes and periodontitis?

For example, if you inform everyone, then circle 10. If you inform some but not all, then chose a number which represents the proportion that you inform.

0 1 2 3 4 5 6 7 8 9 10

Q8.
Thinking of **your last 10 patients with diabetes who you had a conversation with regarding their poor HbA1c levels (i.e. > 58 mmol/mol or > 7.5%).**

How many have you ssuggested to go for a dental check-up?

For example, if you suggest it to everyone, then circle 10. If you inform some but not all, then chose a number which represents the proportion that you inform.

0 1 2 3 4 5 6 7 8 9 10

Q9.
This question refers to **informing your patients with diabetes about the links between diabetes and periodontitis, during a conversation regarding their poor HbA1c levels (i.e. > 58 mmol/mol or > 7.5%).**

For each statement please **read the stem carefully first**, then select an answer that best suits your experience using the options.

For example, take a look at the stem and the first statement below and read them carefully. If you feel confident that you can inform your patient about the links between diabetes and periodontitis even when work is busy, then choose option 'Strongly Agree' or 'Agree'. If you feel that work being busy may affect your confidence in informing your patient about the links, then choose option 'Disagree' or 'Strongly Disagree'. If you are not sure, choose 'Neither agree nor disagree'. Now re-read the stem followed by the next statement, and so on.

Stem: I am confident that I can inform my patients with diabetes about the links between diabetes and periodontitis EVEN WHEN...

Statements:

	Strongly Disagree	Disagree	Neither Agree nor Disagree	Agree	Strongly Agree
... work is busy	○	○	○	○	○
... it is someone else's responsibility	○	○	○	○	○
... there is already too much to do	○	○	○	○	○
... it is not a priority for me	○	○	○	○	○
... it is not a priority for the patient	○	○	○	○	○
... I am not set up for it	○	○	○	○	○
... there are problems accessing dental services	○	○	○	○	○
... I am running late	○	○	○	○	○
... the patient has very few of their own teeth	○	○	○	○	○
... the patient seems to have good dental health	○	○	○	○	○
... my colleagues are doing the same	○	○	○	○	○
... my colleagues are not doing the same	○	○	○	○	○

Q10.
This question refers to **informing your patients with diabetes about the links between diabetes and periodontitis, during a conversation regarding their poor HbA1c levels (i.e. > 58 mmol/mol or > 7.5%).**

Stem: I am confident **that I can provide a suggestion to go for a dental check-up *EVEN* WHEN...**

Statements:

	Strongly Disagree	Disagree	Neither Agree nor Disagree	Agree	Strongly Agree
... work is busy	○	○	○	○	○
... it is someone else's responsibility	○	○	○	○	○
... there is already too much to do	○	○	○	○	○
... it is not a priority for me	○	○	○	○	○
... it is not a priority for the patient	○	○	○	○	○

... I am not set up for it	○	○	○	○	○
... there are problems accessing dental services	○	○	○	○	○
... I am running late	○	○	○	○	○
... the patient has very few of their own teeth	○	○	○	○	○
... the patient seems to have good dental health	○	○	○	○	○
... my colleagues are doing the same	○	○	○	○	○
... my colleagues are not doing the same	○	○	○	○	○

Q11.

This question is about **informing your <u>poorly controlled patients with diabetes</u> (HbA1c > 58 mmol/mol or > 7.5%) about the links between diabetes and periodontitis.**

Please read the statements carefully and chose an appropriate option.

OPTIONS

	Strongly agree	Agree	Neither Agree nor Disagree	Disagree	Strongly Disagree	Not relevant to my role	Not relevant at this stage	Not relevant to diabetes care
Statement 1: I can see how informing patients about the links between diabetes and periodontitis differs from my usual way of working	○	○	○	○	○	○	○	○
Statement 2: Staff in this organisation have a shared understanding of the purpose of informing patients about the links between diabetes and periodontitis	○	○	○	○	○	○	○	○
Statement 3: I understand how informing patients about the links between diabetes and periodontitis affects the nature of my own work	○	○	○	○	○	○	○	○
Statement 4: I can see the potential value of informing patients about the links between diabetes and periodontitis for my work	○	○	○	○	○	○	○	○
Statement 5: I believe that informing patients about the links between diabetes and periodontitis is a legitimate part of my role	○	○	○	○	○	○	○	○

Q12.

This question relates to **suggesting that your patients with HbA1c > 58 mmol/mol or > 7.5%, should go for a dental check-up.**

Please read the statements carefully and chose an appropriate option.

OPTIONS

	Strongly agree	Agree	Neither Agree nor Disagree	Disagree	Strongly Disagree	Not relevant to my role	Not relevant at this stage	Not relevant to diabetes care
Statement 1: I can see how suggesting that your patients with HbA1c > 58 mmol/mol or > 7.5%, should go for a dental check-up differs from my usual way of working	○	○	○	○	○	○	○	○
Statement 2: Staff in this organisation have a shared understanding of the purpose of suggesting that your patients with HbA1c > 58 mmol/mol or > 7.5%, should go for a dental check-up	○	○	○	○	○	○	○	○
Statement 3: I understand how suggesting that your patients with HbA1c > 58 mmol/mol or > 7.5%, should go for a dental check-up affects the nature of my own work	○	○	○	○	○	○	○	○
Statement 4: I can see the potential value of suggesting								

For peer review only - http://bmjopen.bmj.com/site/about/guidelines.xhtml

that your patients with HbA1c > 58 mmol/mol or > 7.5%, should go for a dental check-up for my work	○	○	○	○	○	○	○	○
Statement 5: I believe that suggesting that your patients with HbA1c > 58 mmol/mol or > 7.5%, should go for a dental check-up is a legitimate part of my role	○	○	○	○	○	○	○	○

Q13.
Informing them about the links between diabetes and periodontitis will help my poorly controlled patients with diabetes (HbA1c > 58 mmol/mol or > 7.5%)?

Not at all	A little	Moderately	Quite a bit	Extremely
○	○	○	○	○

Q14.
Suggesting that they go for a dental check-up will help my patients with poorly controlled diabetes (HbA1c > 58 mmol/mol or > 7.5%)?

Not at all	A little	Moderately	Quite a bit	Extremely
○	○	○	○	○

Q15.
Iinforming my patients with poorly controlled diabetes (HbA1c > 58 mmol/mol or > 7.5%) about the links between diabetes and periodontitis, will be a good use of my time?

Never	Rarely	Sometimes	Often	All of the Time
○	○	○	○	○

Q16.
Suggesting that my patients with poorly controlled diabetes (HbA1c > 58 mmol/mol or >7.5%) go for a dental check-up will be a good use of my time?

Never	Rarely	Sometimes	Often	All of the Time
○	○	○	○	○

Q17.
This question asks about the **timing** of **informing** patients about the links between diabetes and periodontitis and **suggesting** to have a dental check-up. Please read the question and tick as many options as you like.

OPTIONS:

QUESTIONS:

	**Q1**: When is a good time to inform patients about the links between diabetes and periodontitis?	**Q2**: When is a good time to suggest having a dental check-up?
Never	☐	☐
At time of periodontal diagnosis	☐	☐
At time of diabetes diagnosis	☐	☐
At their routine dental check-up appointment	☐	☐
Alongside discussion regarding their HbA1c	☐	☐
When the patient has poorly controlled diabetes	☐	☐
When the patient is facing being prescribed oral medication (or additional oral medication) for their diabetes	☐	☐
When the patient is facing being put onto insulin or other injectables	☐	☐
At their first appointment in the practice	☐	☐
When the periodontal condition deteriorates	☐	☐
Other	☐	☐

For peer review only - http://bmjopen.bmj.com/site/about/guidelines.xhtml

Version 1.0

Q18.
This question asks about the **timing** of **informing** patients about the links between diabetes and periodontitis and **suggesting** to have a dental check-up. Please read the question and tick as many options as you like.

	OPTIONS - choose as many as you like							
	No one	GP	GP with special interest in diabetes	Dietician	Diabetologist	Dentist	Dental hygienist/therapist	Other
Q1: Who **could** inform about the links between diabetes and periodontitis?	☐	☐	☐	☐	☐	☐	☐	☐
Q2: Who **should** inform about the links between diabetes and periodontitis?	☐	☐	☐	☐	☐	☐	☐	☐
Q3: Who **could** suggest having a dental check-up?	☐	☐	☐	☐	☐	☐	☐	☐
Q4: Who **should** suggest having a dental check-up?	☐	☐	☐	☐	☐	☐	☐	☐

Q19.
This question relates to **your next 10 patients with poorly controlled diabetes (HbA1c>58 mmol/mol or > 7.5%).**

How many do you intend to inform about the links between diabetes and periodontitis?

0 1 2 3 4 5 6 7 8 9 10

Q20.
This question relates to **your next 10 patients with poorly controlled diabetes (HbA1c>58 mmol/mol or > 7.5%).**

How many do you intend to suggest going for a dental check-up?

0 1 2 3 4 5 6 7 8 9 10

Q21. Please use this space to clarify any responses or write any comments, if you would like to.

Reporting checklist for cross sectional study.

Based on the STROBE cross sectional guidelines.

Instructions to authors

Complete this checklist by entering the page numbers from your manuscript where readers will find each of the items listed below.

Your article may not currently address all the items on the checklist. Please modify your text to include the missing information. If you are certain that an item does not apply, please write "n/a" and provide a short explanation.

Upload your completed checklist as an extra file when you submit to a journal.

In your methods section, say that you used the STROBE cross sectionalreporting guidelines, and cite them as:

von Elm E, Altman DG, Egger M, Pocock SJ, Gotzsche PC, Vandenbroucke JP. The Strengthening the Reporting of Observational Studies in Epidemiology (STROBE) Statement: guidelines for reporting observational studies.

		Reporting Item	Page Number
**Title and abstract**			
Title	#1a	Indicate the study's design with a commonly used term in the title or the abstract	1

For peer review only

Abstract	#1b	Provide in the abstract an informative and balanced summary of what was done and what was found	2
**Introduction**			
Background / rationale	#2	Explain the scientific background and rationale for the investigation being reported	5
Objectives	#3	State specific objectives, including any prespecified hypotheses	6
**Methods**			
Study design	#4	Present key elements of study design early in the paper	7
Setting	#5	Describe the setting, locations, and relevant dates, including periods of recruitment, exposure, follow-up, and data collection	10
Eligibility criteria	#6a	Give the eligibility criteria, and the sources and methods of selection of participants.	10
	#7	Clearly define all outcomes, exposures, predictors, potential confounders, and effect modifiers. Give diagnostic criteria, if applicable	10
Data sources / measurement	#8	For each variable of interest give sources of data and details of methods of assessment (measurement). Describe comparability of assessment methods if there is more than one group. Give information separately for for exposed and unexposed groups if applicable.	10

For peer review only

Bias	#9	Describe any efforts to address potential sources of bias	4
Study size	#10	Explain how the study size was arrived at	11
Quantitative variables	#11	Explain how quantitative variables were handled in the analyses. If applicable, describe which groupings were chosen, and why	11
Statistical methods	#12a	Describe all statistical methods, including those used to control for confounding	11
Statistical methods	#12b	Describe any methods used to examine subgroups and interactions	11
Statistical methods	#12c	Explain how missing data were addressed	12
Statistical methods	#12d	If applicable, describe analytical methods taking account of sampling strategy	12
Statistical methods	#12e	Describe any sensitivity analyses	11
**Results**			
Participants	#13a	Report numbers of individuals at each stage of study—eg numbers potentially eligible, examined for eligibility, confirmed eligible, included in the study, completing follow-up, and analysed. Give information separately for for exposed and unexposed groups if applicable.	12
Participants	#13b	Give reasons for non-participation at each stage	12

For peer review only

Participants	#13c	Consider use of a flow diagram	
Descriptive data	#14a	Give characteristics of study participants (eg demographic, clinical, social) and information on exposures and potential confounders. Give information separately for exposed and unexposed groups if applicable.	13
Descriptive data	#14b	Indicate number of participants with missing data for each variable of interest	12
Outcome data	#15	Report numbers of outcome events or summary measures. Give information separately for exposed and unexposed groups if applicable.	15
Main results	#16a	Give unadjusted estimates and, if applicable, confounder-adjusted estimates and their precision (eg, 95% confidence interval). Make clear which confounders were adjusted for and why they were included	15
Main results	#16b	Report category boundaries when continuous variables were categorized	15
Main results	#16c	If relevant, consider translating estimates of relative risk into absolute risk for a meaningful time period	n/a
Other analyses	#17	Report other analyses done—e.g., analyses of subgroups and interactions, and sensitivity analyses	15
**Discussion**			
Key results	#18	Summarise key results with reference to study objectives	19

For peer review only

Limitations	#19	Discuss limitations of the study, taking into account sources of potential bias or imprecision. Discuss both direction and magnitude of any potential bias.	4
Interpretation	#20	Give a cautious overall interpretation considering objectives, limitations, multiplicity of analyses, results from similar studies, and other relevant evidence.	20
Generalisability	#21	Discuss the generalisability (external validity) of the study results	4
**Other Information**			
Funding	#22	Give the source of funding and the role of the funders for the present study and, if applicable, for the original study on which the present article is based	21

The STROBE checklist is distributed under the terms of the Creative Commons Attribution License CC-BY. This checklist was completed on 13. June 2019 using https://www.goodreports.org/, a tool made by the EQUATOR Network in collaboration with Penelope.ai

For peer review only
